# Supplementary material for: Antagonistic activities of CDC14B and CDK1 on USP9X regulate WT1-dependent mitotic transcription and survival
Source: Nat Commun. 2020 Mar 9;11:1268. doi: 10.1038/s41467-020-15059-5 (PMC7063047; doi:10.1038/s41467-020-15059-5)
Supplement: Supplementary file 1 — Supplementary Information [file 41467_2020_15059_MOESM1_ESM.pdf]

Antagonistic activities of CDC14B and CDK1 on USP9X regulate WT1-dependent mitotic transcription and survival

Dietachmayr et al.

a

gene	TAP control	TAP CDC14B async	TAP CDC14B G2/M
CDC14B	***0***	***24***	***22***
USP9X	***0***	***4***	***7***
RPL3	0	2	3
RPL17	0	0	3
RPL8	0	0	2
RPL23A	0	2	2
RPS32	0	0	2

b

Ratio H/L normalized	Position	Peptides	Gene name
4,4188	2563	3	USP9X
	1226	1	USP9X

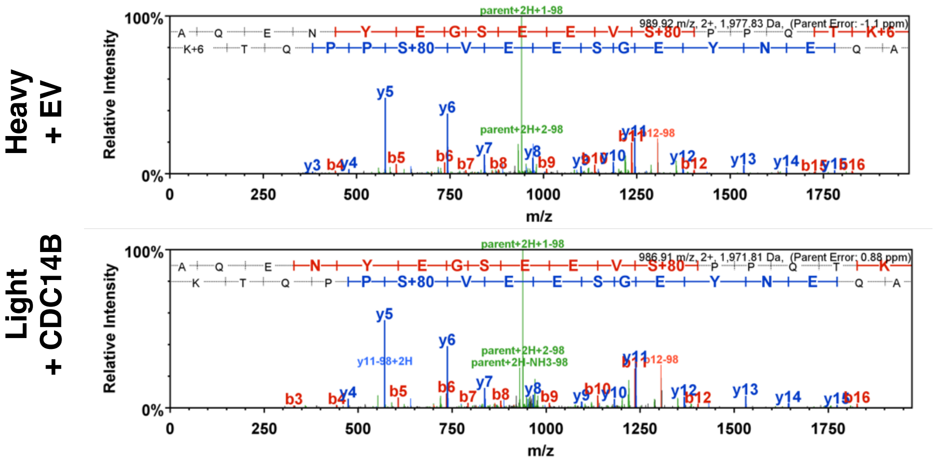

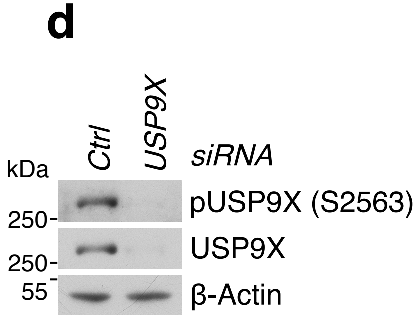

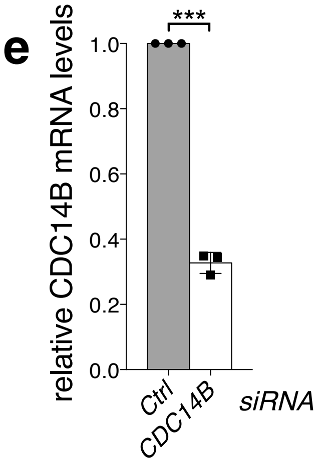

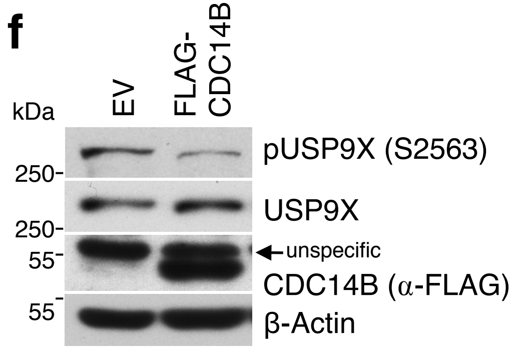

g

CDK1 recognition motif

USP9X protein: (2557) G S E E V S P P Q T K D Q Stop

CDK1 Consensus motif: S/T P

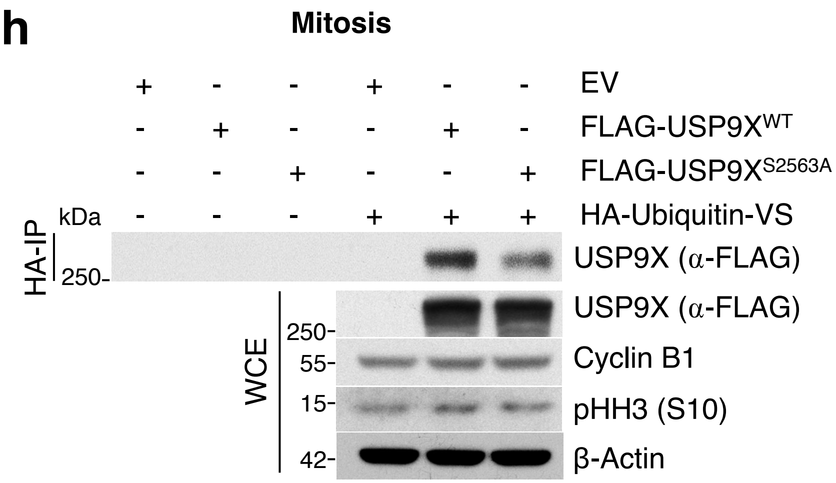

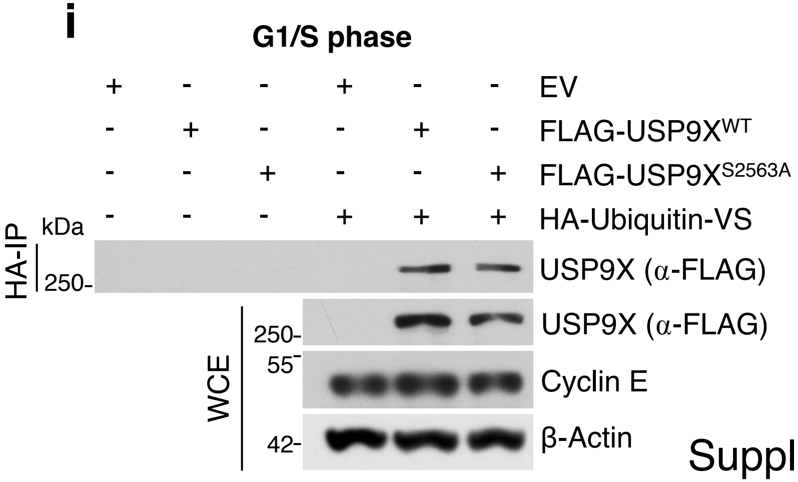

Supplementary Fig. 1

Supplementary Figure 1. Opposing CDK1/CDC14B phosphorylation of USP9X at Serine 2563 regulates its DUB activity

- **a.** Mass spectrometric analysis of the CDC14B interactome following tandem purification (TAP) of CDC14B from either asynchronous (AS) or G2/M-arrested HEK 293T cells. Hits with most significant changes between G2/M and asynchronous cells are shown. Numbers of unique peptides are indicated.
- **b.** CDC14B-dependent phosphorylation of USP9X at Serine 2563 by mass spectrometric phospho-analysis of SILAC-labeled HEK 293T cells following overexpression of CDC14B or control vector. Control cells (EV = expression vector) were labeled with heavy ("H"), CDC14B-overexpressing cells with light ("L") SILAC medium.
- **c.** Mass spectra of the USP9X (Serine 2563) phosphopeptides as described in **b**.
- **d.** Validation of pUSP9X (S2563) antibody by immunoblot following transfection with either control or *USP9X* siRNA in U2OS cells.
- **e.** Quantitative RT-PCR of CDC14B mRNA confirming knockdown efficiency in mitotic U2OS cells after treatment with control or *CDC14B* siRNA. Mean and standard deviations as error bars are shown from n=3 biologically independent experiments. One sample t-test was applied with ***p=0.0008.
- **f.** Immunoblot analysis showing hypo-phosphorylation of USP9X at Serine 2563 in HeLa cells overexpressing CDC14B. FLAG-CDC14B or control vector overexpressing HeLa cells were subjected to Western Blot with the indicated antibodies.
- **g.** Homology between the C terminus of the USP9X protein and the canonical minimal CDK1 recognition site (S/T – P).

h. *In vitro* deubiquitylation assay showing decreased mitotic activity of USP9X^{S2563A} compared to USP9X^{WT}. HEK 293T cells were transfected with FLAG-USP9X^{WT}, FLAG-USP9X^{S2563A} or a control vector (EV) as indicated and arrested in mitosis using nocodazole. Cells were collected and lysed (WCE), then lysates were divided and incubated with recombinant HA-Ubiquitin-Vinyl sulfone or solvent. HA immunoprecipitation was performed and immunoprecipitated protein detected by Western Blot.

i. *In vitro* deubiquitylation assay showing comparable S phase activity of USP9X^{S2563A} compared to USP9X^{WT}. HEK 293T cells were transfected with FLAG-USP9X^{WT}, FLAG-USP9X^{S2563A} or a control vector (EV) as indicated and arrested in G1/S phase using Hydroxyurea. Cells were then processed as in **h**.

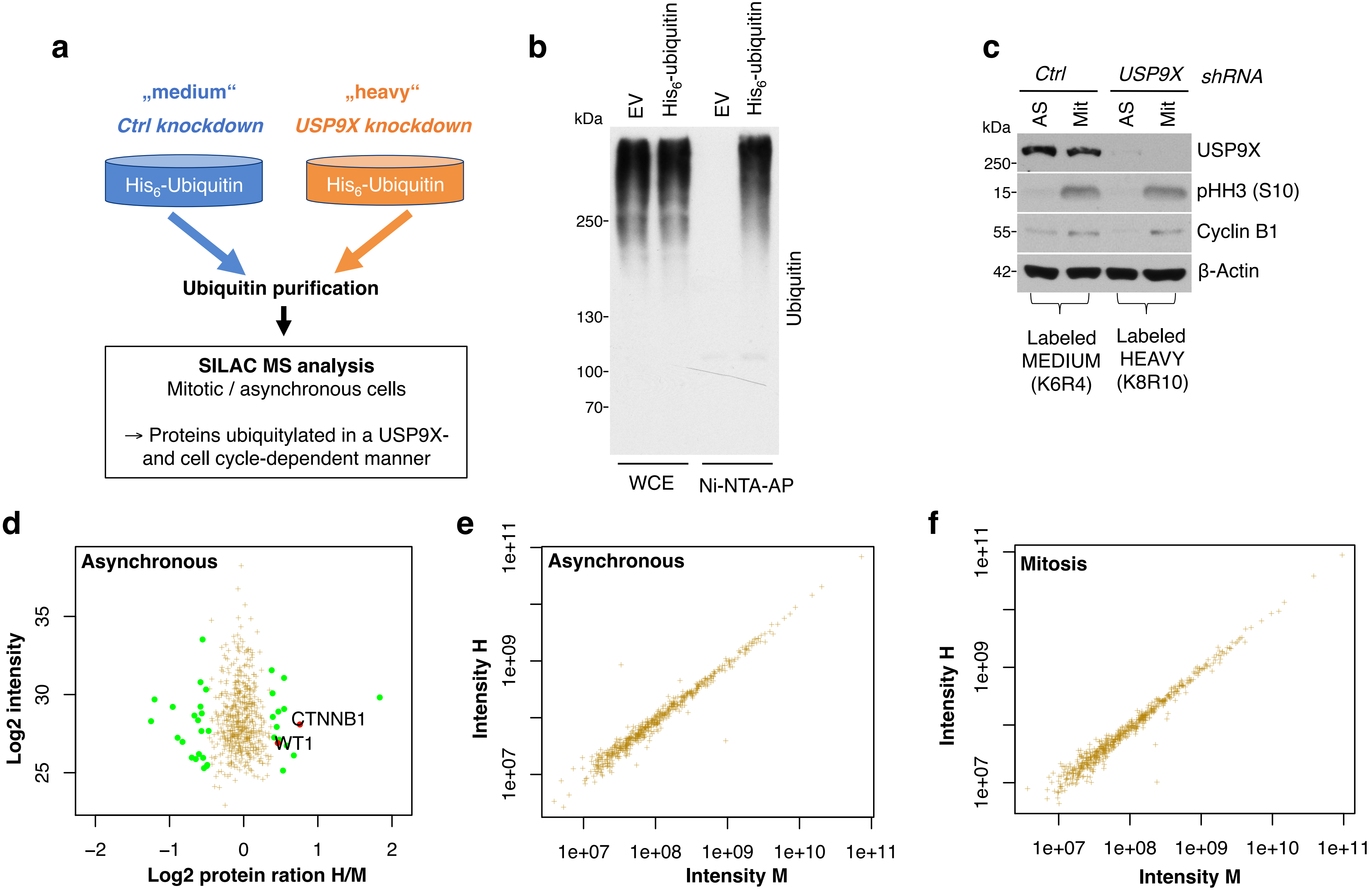

Supplementary Fig. 2

Supplementary Figure 2. USP9X-dependent ubiquitome analysis reveals WT1 as a mitotic USP9X substrate

a. Schematic of a screening approach for cell cycle-dependent USP9X substrates. The indicated SILAC medium was added to either control- or *USP9X*-depleted, stably His_6-ubiquitin-expressing HEK 293T cells. Following ubiquitin purification, differentially ubiquitylated proteins were analysed by mass spectrometry. The experiment was performed separately in asynchronous or mitotically arrested cells.

b. Immunoblot analysis from stably ubiquitin-expressing HEK 293T cells confirming physiological levels of ubiquitin in whole cell extracts. Cells were transduced with either control vector (EV) or a His_6-ubiquitin-carrying construct and selected for expression. Cells were lysed under denaturing conditions and Ni-NTA precipitation was performed followed by Western Blot analysis.

c. Immunoblot analysis showing *USP9X* depletion and mitotic synchronization of HEK 293T cells used for ubiquitome analysis. Stably His_6-ubiquitin-expressing cells were transfected with control or *USP9X* shRNA and either left asynchronous (AS) or synchronised in mitosis using nocodazole (Mit). Cells were grown in medium (“M”) or heavy (“H”) SILAC medium as indicated and proteasomal inhibition with bortezomib was applied before collection.

d. Mass spectrometric analysis of the USP9X-dependent ubiquitome in asynchronous HEK 293T cells. *USP9X* knockdown cells were cultured in SILAC media with heavy-labeled aminoacids ("H"). Control knockdown cells were cultured in SILAC media containing medium-labeled aminoacids ("M").

e. Intensity values of analysed peptides shown in **d.** under *USP9X* knockdown conditions ("intensity H") and control knockdown conditions ("intensity M").

f. Intensity values of analysed peptides shown in Fig. 2a under *USP9X* knockdown conditions ("intensity H") and control knockdown conditions ("intensity M").

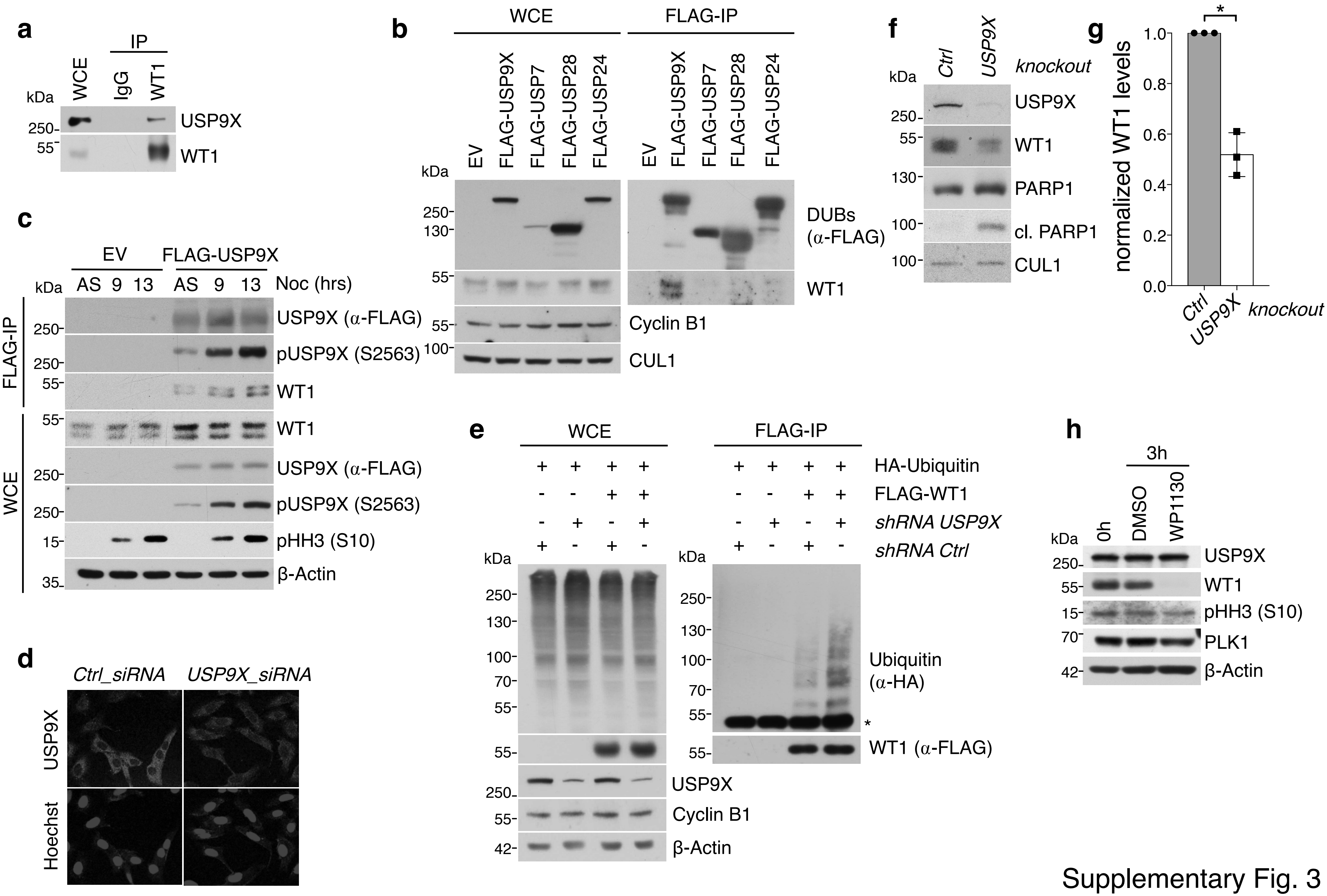

Supplementary Fig. 3

Supplementary Figure 3. WT1 is a direct substrate of USP9X in mitosis

a. Co-immunoprecipitation of endogenous WT1 and endogenous USP9X. HEK 293T cells were synchronized in mitosis using nocodazole, lysed, subjected to IgG or anti-WT1 antibody immunoprecipitation and analysed by Western Blot.

b. Co-immunoprecipitation of USP9X with WT1 in HEK 293T cells that were transfected with the indicated FLAG-tagged deubiquitinases before synchronisation in mitosis (EV = expression vector). Cells were lysed, subjected to FLAG-immunoprecipitation and analysed by Western Blot.

c. Mitotic co-immunoprecipitation of USP9X and WT1 from HEK 293T cells that were transfected with either FLAG-USP9X or a control vector (EV) and either kept asynchronous (AS) or treated with nocodazole. Western Blot of whole cell extracts and immunoprecipitated proteins.

d. Validation of the USP9X antibody for immunostaining by indirect immunofluorescence of U2OS cells that were either treated with *Usp9X* siRNA or control siRNA and subsequently stained in a sequential manner with a USP9X specific antibody and an AlexaFluor488 secondary antibody. Hoechst staining identifies DNA.

e. USP9X-dependent *in vivo* ubiquitylation of WT1 in HEK 293T cells that were transfected with control or *USP9X* shRNA-containing plasmid, FLAG-tagged WT1 and HA-tagged ubiquitin and synchronized in mitosis using nocodazole. 14 hours before collection bortezomib was added. Cells were then lysed under denaturing conditions and FLAG-immunopurification was performed. Western Blot analysis of whole cell extracts (WCE; left) and

immunoprecipitated WT1 (right) is shown. * indicates immunoglobulin heavy chain detected by the secondary WB antibody.

f. Immunoblot analysis of USP9X-dependent WT1 stability in CRISPR/Cas9 mediated control or *USP9X* knockout U2OS cells that were treated with nocodazole for synchronisation in mitosis and subjected to Western Blot.

g. Quantification of WT1 destabilisation in response to *USP9X* knockout in n=3 biologically independent experiments performed as in **e**. Western Blot bands were quantified using ImageJ software and normalized to loading control (CUL1 or β-actin). Mean and standard deviations as error bars are shown. Ratio paired t-test was applied with *p=0.0204.

h. Immunoblot analysis of WT1 stability under USP9X inhibition with WP1130. U2OS cells were synchronized in mitosis using nocodazole. Mitotic cells were either collected (0h), or treated with DMSO or WP1130 for 3h. Together with DMSO or WP1130, Z-VAD-FMK was added. Lysates were analysed by Western Blot.

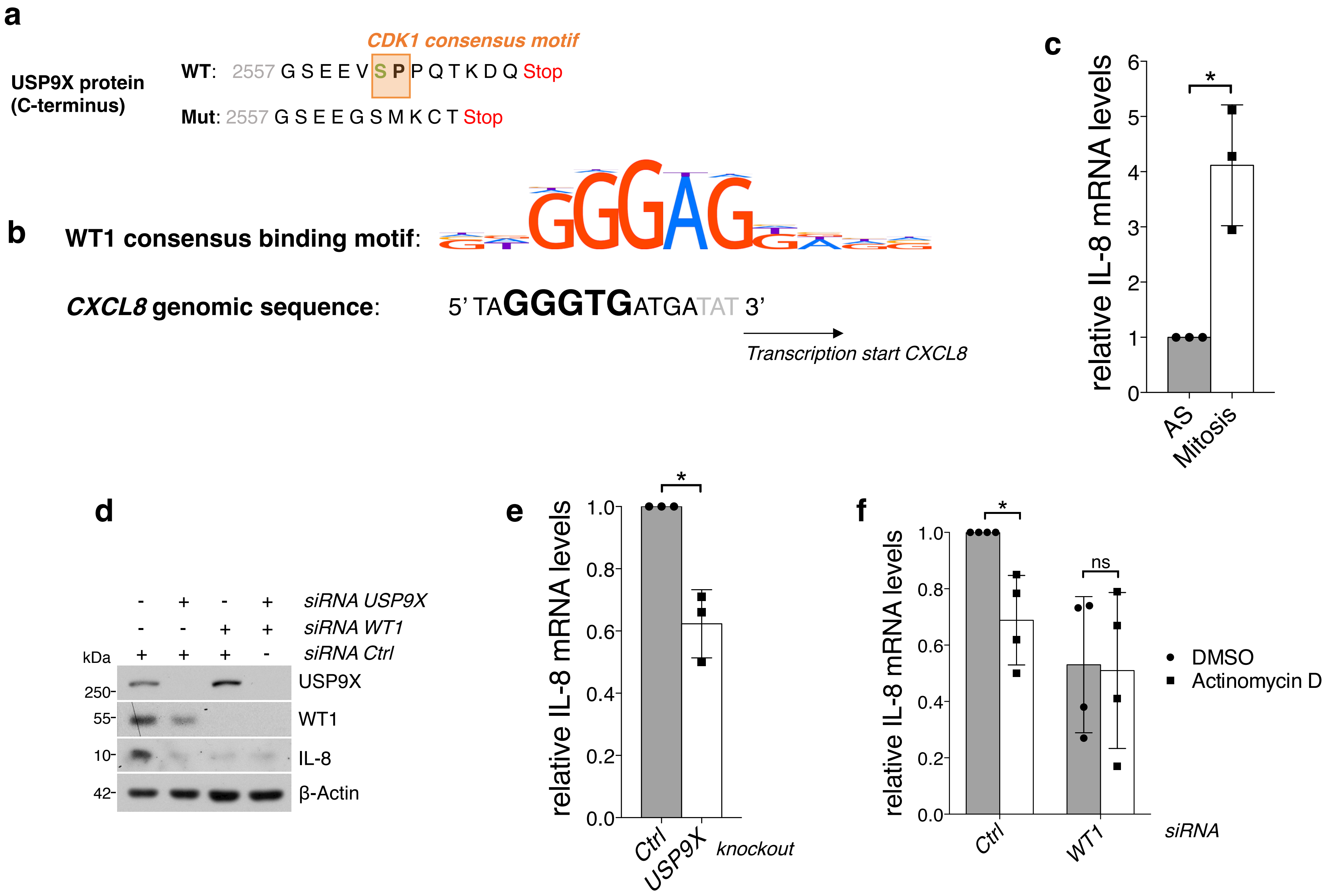

Supplementary Fig. 4

Supplementary Figure 4. IL-8 is transcriptionally regulated by WT1 and pUSP9X (S2563)

- **a.** CRISPR/Cas9-mediated homozygous disruption of the CDK1 recognition motif in USP9X generates USP9X^{Mut} U2OS cells (bottom line). For comparison, the C-terminus of the wildtype USP9X protein (USP9X^{WT}) is displayed.
- **b.** Position weight matrix of the WT1 binding motif according to the Homo sapiens COmprehensive MOdel Collection (HOCOMOCO) web tool (http://hocomoco11.autosome.ru) corresponding to the genomic sequence at the transcriptional start site of the *CXCL8* gene.
- **c.** Quantitative RT-PCR for IL-8 mRNA in U2OS cells either left asynchronous or synchronized in mitosis using double thymidine block and collection by mitotic shake-off after the second thymidine release. Mean and standard deviations as error bars are shown from n=3 biologically independent experiments. One sample t-test was applied with *p=0.0387.
- **d.** Immunoblot analysis confirming WT1- and USP9X-dependent IL-8 expression in U2OS cells that were transfected with control, *USP9X*, *WT1* or *USP9X* and *WT1* siRNA and arrested in mitosis using nocodazole. Cells were collected, analysed by Western Blot with the respective antibodies and by quantitative RT-PCR for IL-8 mRNA (see Fig. 3f).
- **e.** Quantitative RT-PCR in mitotic U2OS cells with control or *USP9X* knockout confirming USP9X-dependent IL-8 expression. Mean and standard deviations as error bars are shown from n=3 biologically independent experiments. One sample t-test was applied with *p=0.0271.

f. WT1-dependent IL-8 transcription in mitotic U2OS cells that were transfected with control or *WT1* siRNA. After 15 hours nocodazole treatment, mitotic shake-off was performed. Mitotic cells of each condition were kept in nocodazole and simultaneously treated with either DMSO or actinomycin D for three hours and then analysed for IL-8 mRNA by quantitative PCR. Mean and standard deviations as error bars are shown from n=4 biologically independent experiments. One sample t-test (control siRNA) and ratio paired t-test (*WT1* siRNA) were applied with *p(*Ctrl* siRNA)=0.0293, p(*WT1* siRNA)=0.478.

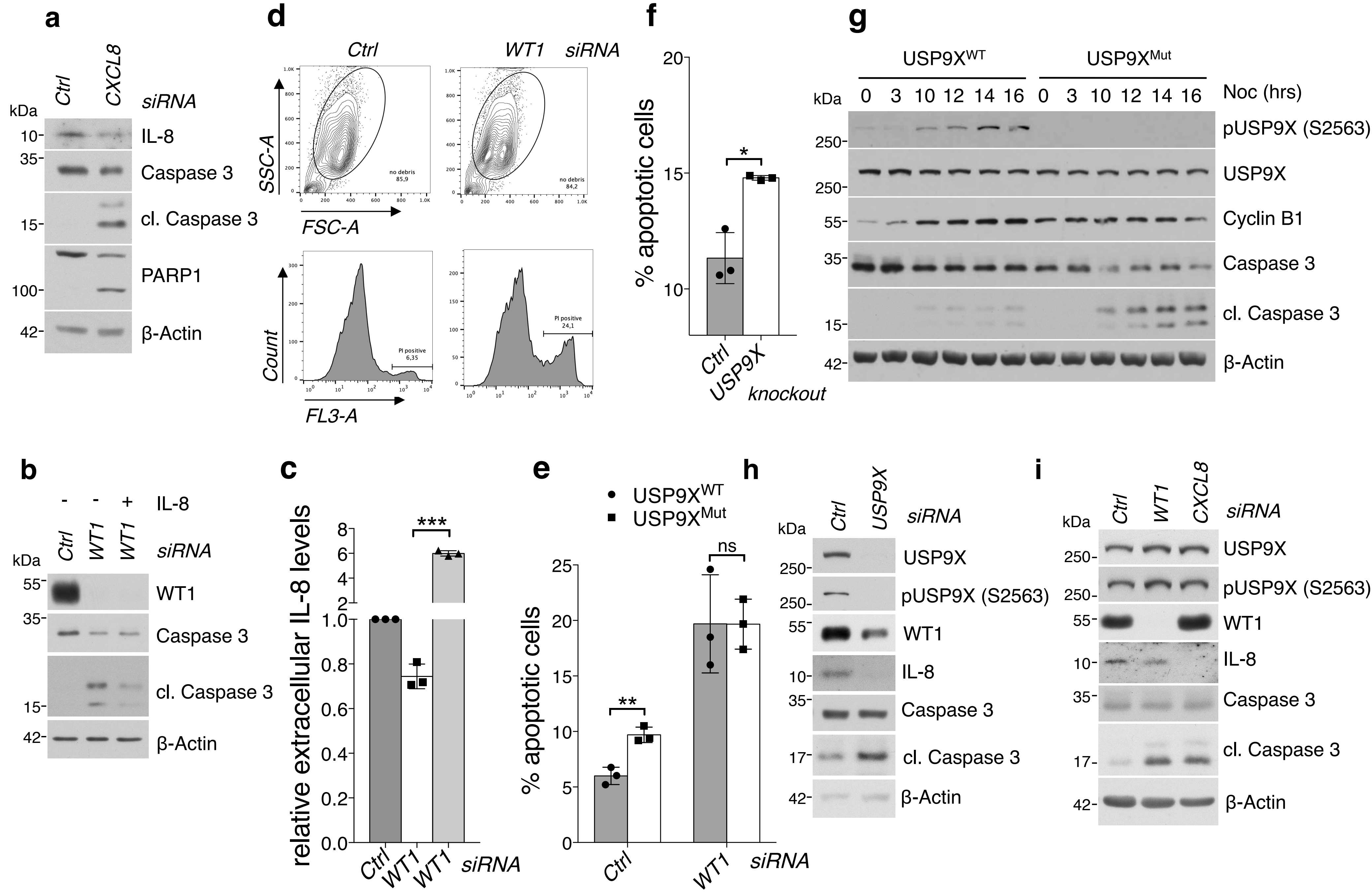

Supplementary Fig. 5

Supplementary Figure 5. CDC14B/CDK1-regulated pUSP9X (S2563) prevents mitotic apoptosis via WT1 and IL-8

- **a.** Immunoblot analysis of U2OS cells showing IL-8 dependence of mitotic apoptosis following a double thymidine block and release to mitosis.
- **b.** Immunoblot analysis confirming reversal of mitotic apoptosis following exogenous reconstitution of IL-8 in WT1-depleted cells. Experiment was performed as in Fig. 4d with addition of exogenous IL-8 for the last 48 hours. Cells were treated with nocodazole for 8 hours.
- **c.** Reconstitution of extracellular IL-8 levels measured by ELISA in n=3 biologically independent experiments performed as in **b**. Mean and standard deviations are shown, ratio paired t-test was applied with ***p=0.0002.
- **d.** Flow cytometry and gating strategy to determine apoptotic cell death in Fig. 4h and Supplementary Figures 5e,f. Cell debris was gated out ("intact cells", upper panel) and FL3 high cells were quantified to determine PI positivity and therefore apoptotic cell death (lower panel). Control and *WT1* knockdown samples from the quantifications in Supplementary Fig. 5e are shown.
- **e.** Induction of mitotic apoptosis in $USP9X^{WT}$ and $USP9X^{Mut}$ cells following control or *WT1* knockdown. U2OS cells were treated as in Fig. 4g and analysed by flow cytometry. PI-positive cells were quantified in each sample using FlowJo software. Error bars display standard deviations from n=3 biologically independent experiments. Paired t-test was applied with **p(*Ctrl* siRNA)=0.0063; p(*WT1* siRNA)=0.9823.
- **f.** Induction of mitotic apoptosis in *USP9X* knockout versus control U2OS cells that were synchronized in mitosis and stained with propidium iodide. Propidium iodide positive cells were quantified in each sample using FlowJo

software. Mean and standard deviations are shown from n=3 biologically independent experiments. Paired t-test was applied with *p=0.0277.

g. Immunoblot analysis showing induction of mitotic apoptosis in $USP9X^{Mut}$ compared to $USP9X^{WT}$ U2OS cells that were collected at the above given time points of nocodazole exposure for mitotic synchronization.

h. Mitotic apoptosis in response to *USP9X* versus control knockdown in A549 cells that were treated with the respective siRNA and arrested in mitosis using nocodazole for 15 hours. Samples were collected, lysed and analysed by Western Blot.

i. Mitotic apoptosis in response to *CXCL8* or *WT1* versus control knockdown in A549 cells that were treated with the respective siRNA and arrested in mitosis using nocodazole for 8 hours.
