# Supplementary material for: Antagonistic activities of CDC14B and CDK1 on USP9X regulate WT1-dependent mitotic transcription and survival
Source: Nat Commun. 2020 Mar 9;11:1268. doi: 10.1038/s41467-020-15059-5 (PMC7063047; doi:10.1038/s41467-020-15059-5)
Supplement: Supplementary file 4 — Source Data [file 41467_2020_15059_MOESM4_ESM.zip › Source data file I.pdf]

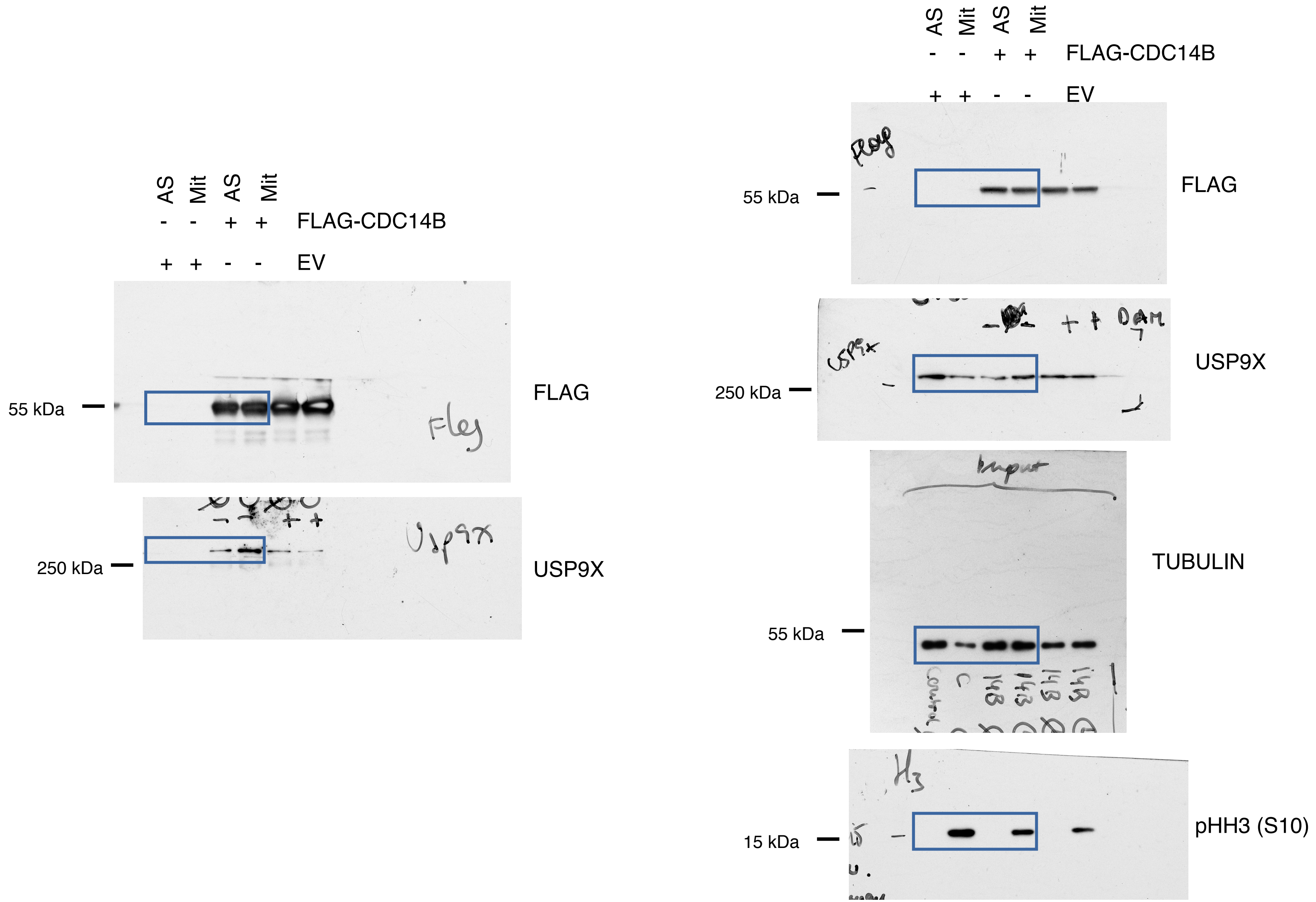

AS Mit AS Mit
- - + + FLAG-CDC14B
+ + - - EV
55 kDa
FLAG
250 kDa
USP9X
AS Mit AS Mit
- - + + FLAG-CDC14B
+ + - - EV
55 kDa
FLAG
250 kDa
USP9X
55 kDa
TUBULIN
15 kDa
pHH3 (S10)

Figure 1a

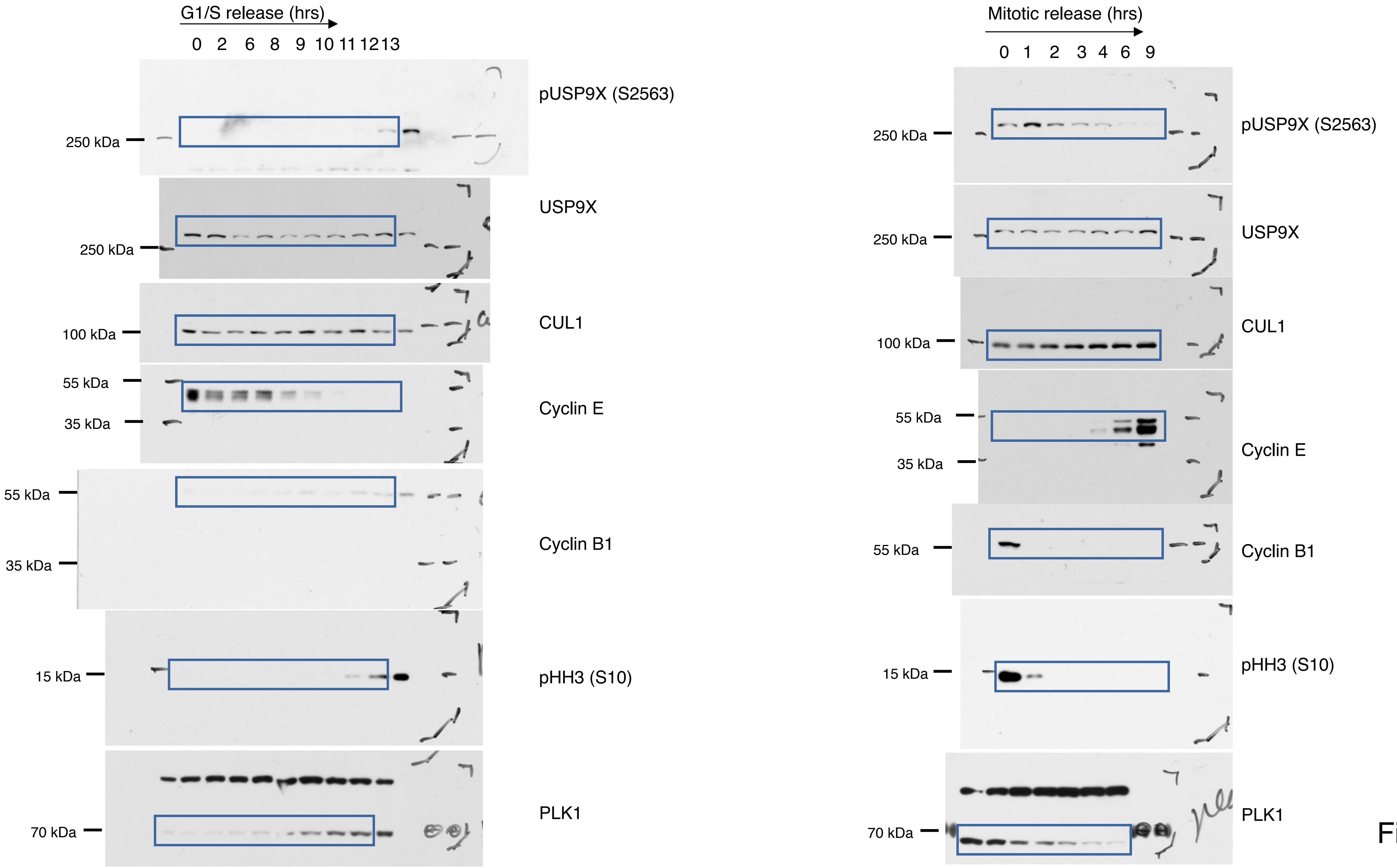
G1/S release (hrs)
0 2 6 8 9 10 11 12 13
pUSP9X (S2563)
250 kDa
USP9X
250 kDa
CUL1
100 kDa
Cyclin E
55 kDa
35 kDa
Cyclin B1
55 kDa
35 kDa
pHH3 (S10)
15 kDa
PLK1
70 kDa
Mitotic release (hrs)
0 1 2 3 4 6 9
pUSP9X (S2563)
250 kDa
USP9X
250 kDa
CUL1
100 kDa
Cyclin E
55 kDa
35 kDa
Cyclin B1
55 kDa
pHH3 (S10)
15 kDa
PLK1
70 kDa

Figure 1b

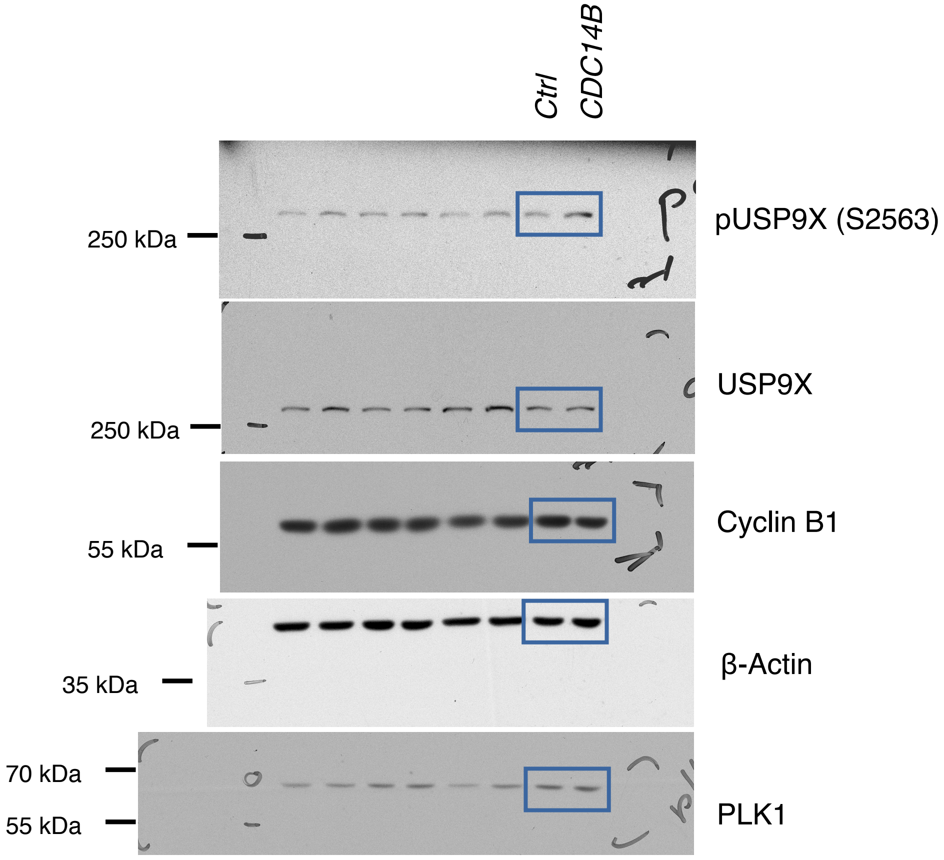
Ctrl
CDC14B
250 kDa
pUSP9X (S2563)
250 kDa
USP9X
55 kDa
Cyclin B1
35 kDa
β-Actin
70 kDa
55 kDa
PLK1

Figure 1c

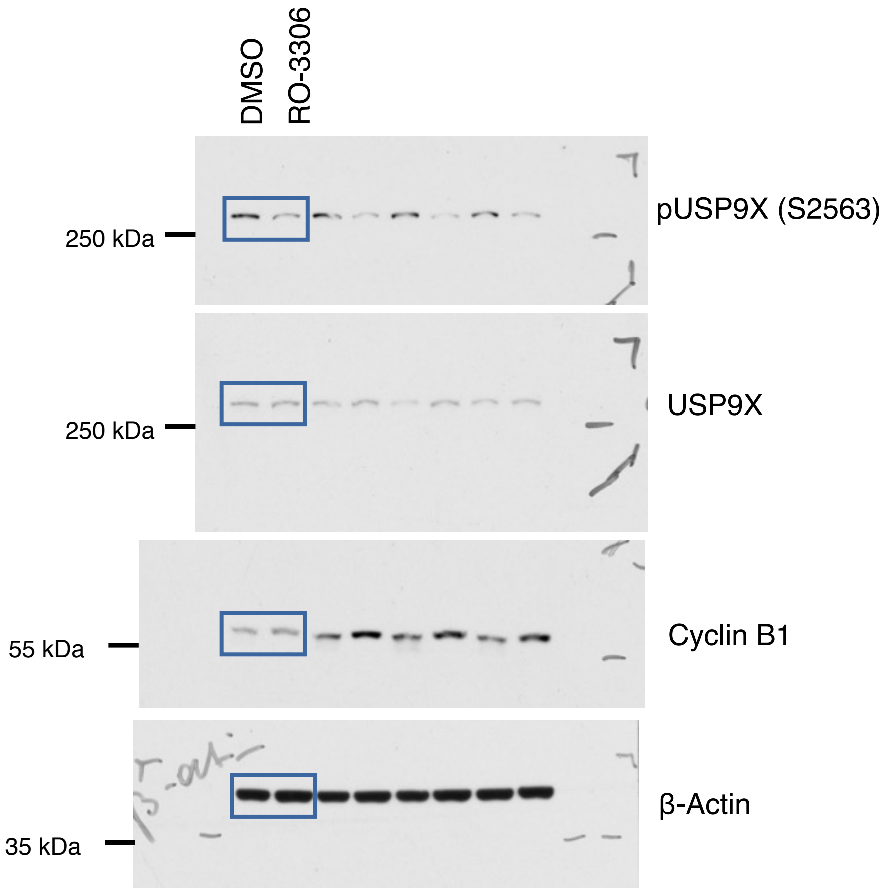

DMSO
RO-3306
pUSP9X (S2563)
USP9X
Cyclin B1
β-Actin
250 kDa
250 kDa
55 kDa
35 kDa

Figure 1e

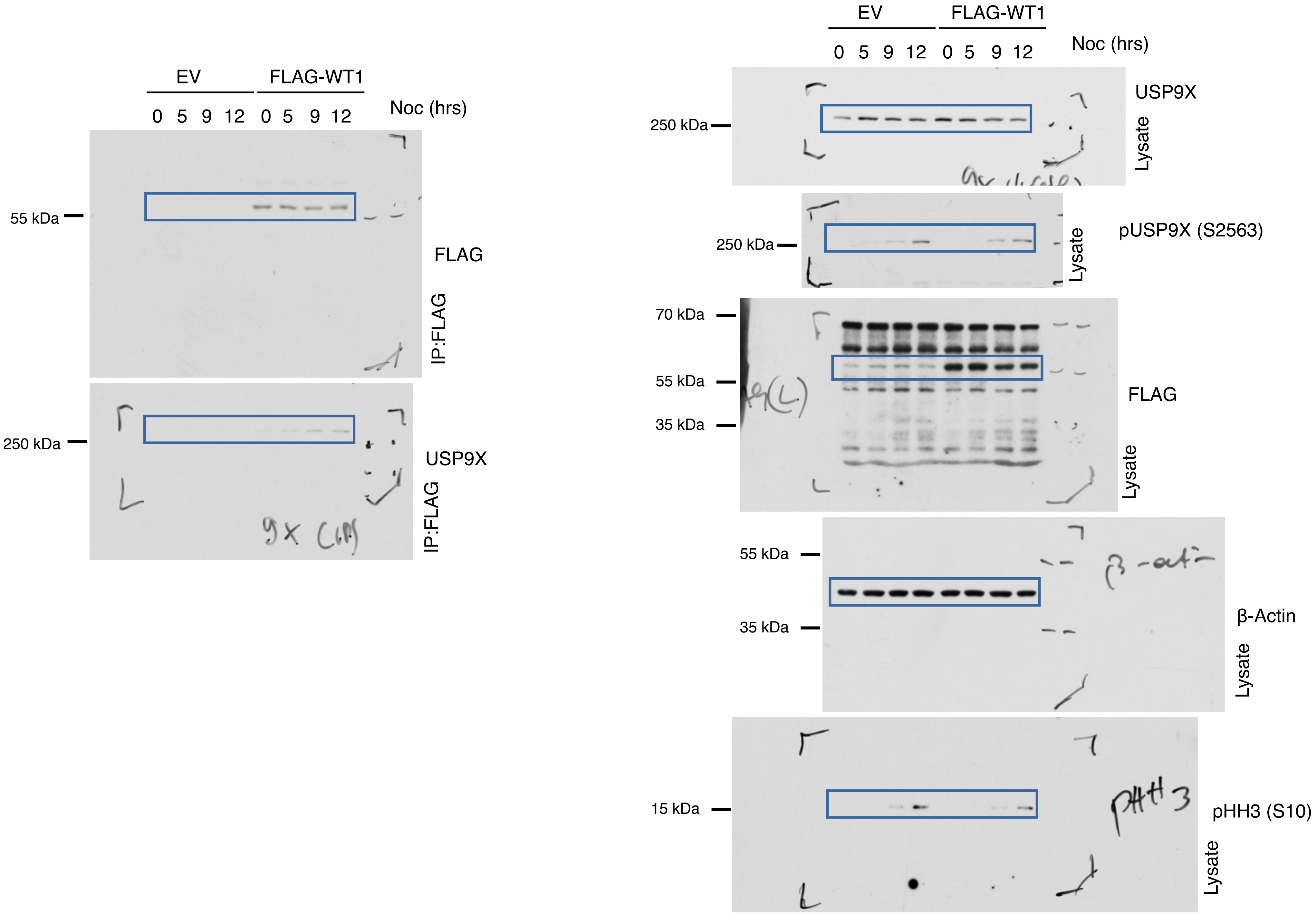
EV
FLAG-WT1
0 5 9 12 0 5 9 12
Noc (hrs)
55 kDa
FLAG
IP:FLAG
250 kDa
USP9X
IP:FLAG
EV
FLAG-WT1
0 5 9 12 0 5 9 12
Noc (hrs)
250 kDa
USP9X
Lysate
250 kDa
pUSP9X (S2563)
Lysate
70 kDa
55 kDa
35 kDa
FLAG
Lysate
55 kDa
35 kDa
β-Actin
Lysate
15 kDa
pHH3 (S10)
Lysate

Figure 2b

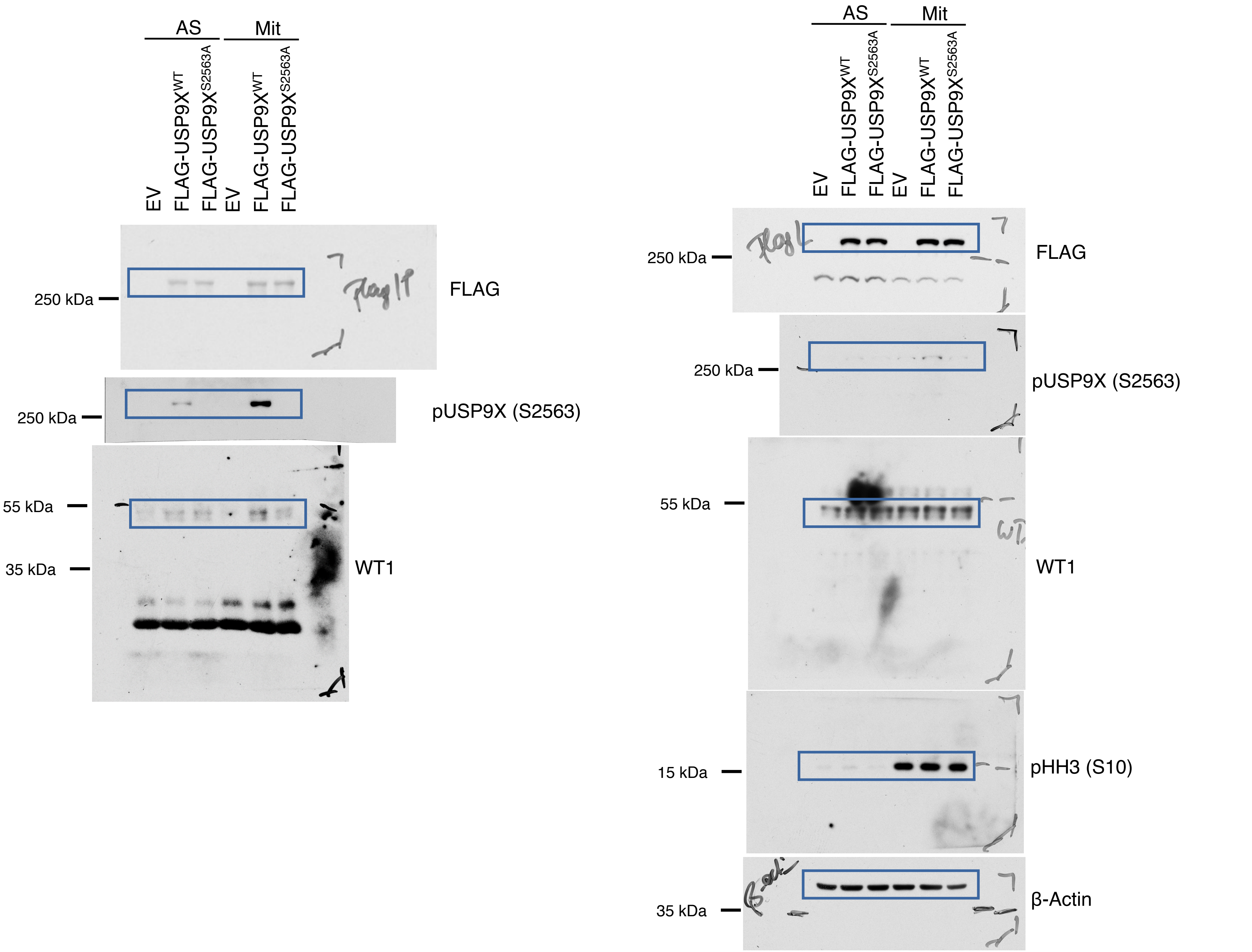
AS
Mit
EV
FLAG-USP9X^{WT}
FLAG-USP9X^{S2563A}
250 kDa
FLAG
pUSP9X (S2563)
55 kDa
35 kDa
WT1
15 kDa
pHH3 (S10)
β-Actin

Figure 2c

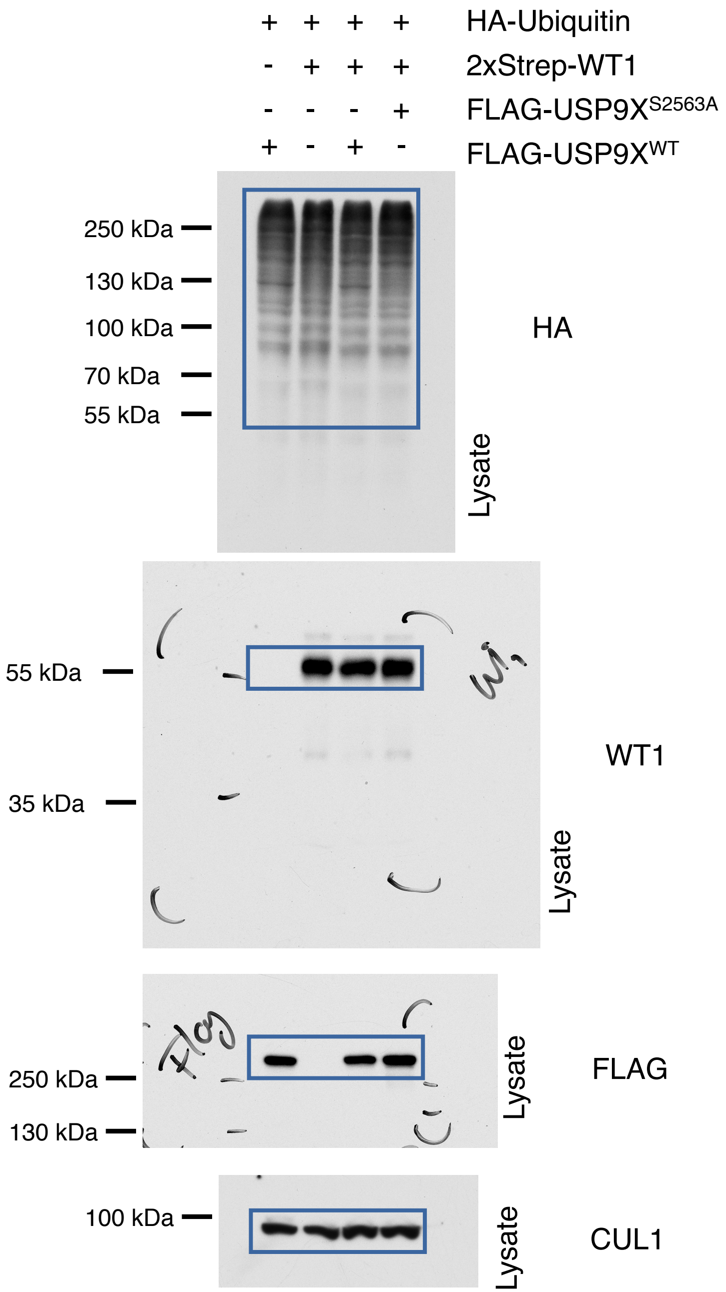

+ + + + HA-Ubiquitin
- + + + 2xStrep-WT1
- - - + FLAG-USP9X S2563A
+ - + - FLAG-USP9X WT
250 kDa
130 kDa
100 kDa
70 kDa
55 kDa
HA
Lysate
55 kDa
35 kDa
WT1
Lysate
250 kDa
130 kDa
FLAG
Lysate
100 kDa
CUL1
Lysate

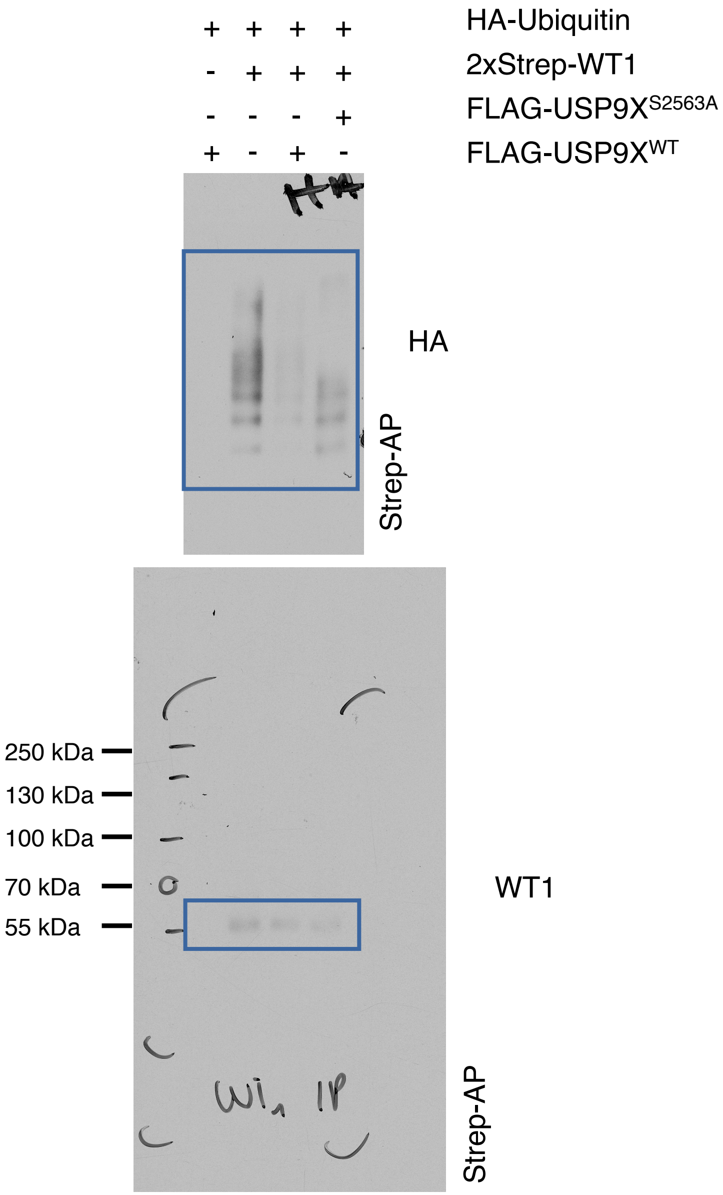

+ + + + HA-Ubiquitin
- + + + 2xStrep-WT1
- - - + FLAG-USP9X S2563A
+ - + - FLAG-USP9X WT
HA
Strep-AP
250 kDa
130 kDa
100 kDa
70 kDa
55 kDa
WT1
Strep-AP

Figure 2f

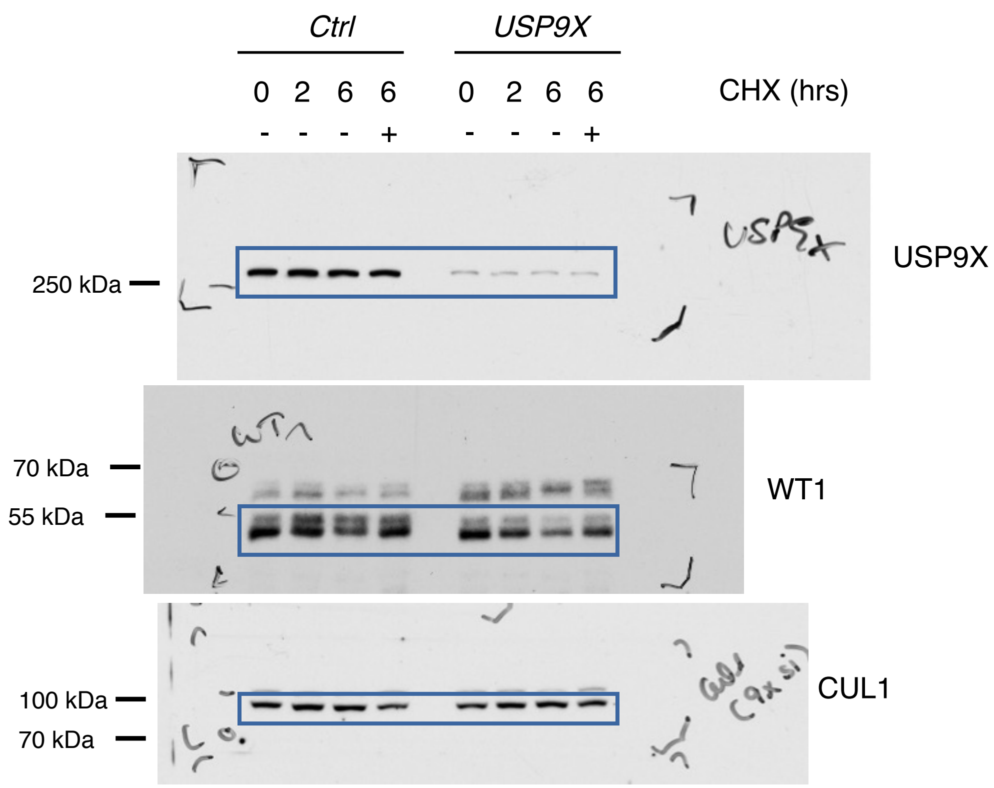
Ctrl
USP9X
0 2 6 6
- - - +
0 2 6 6
- - - +
CHX (hrs)
250 kDa
USP9X
70 kDa
55 kDa
WT1
100 kDa
70 kDa
CUL1

Figure 2g

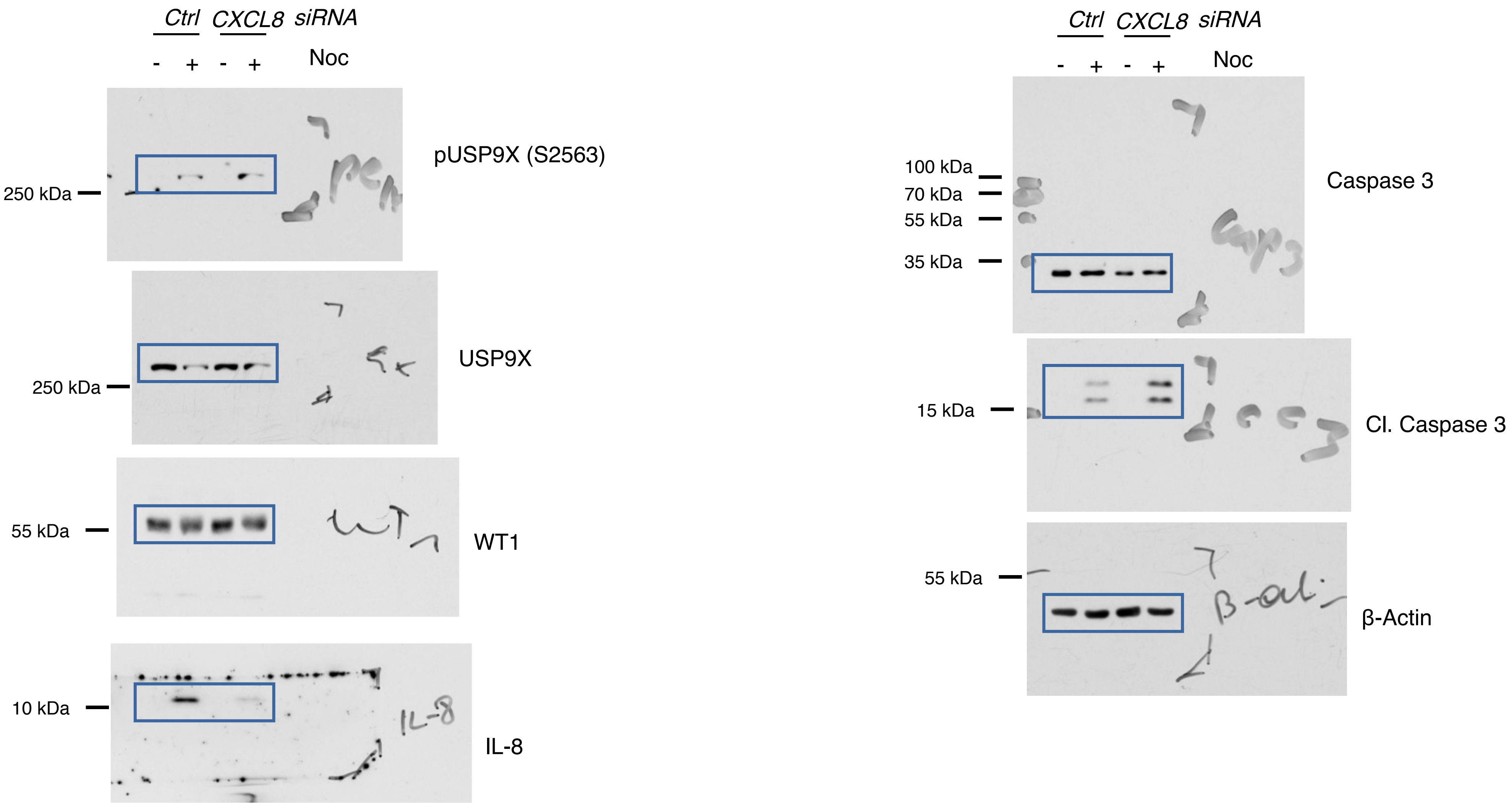

Ctrl CXCL8 siRNA
- + - + Noc
250 kDa
pUSP9X (S2563)
250 kDa
USP9X
55 kDa
WT1
10 kDa
IL-8
Ctrl CXCL8 siRNA
- + - + Noc
100 kDa
70 kDa
55 kDa
35 kDa
Caspase 3
15 kDa
Cl. Caspase 3
55 kDa
β-Actin

Figure 4a

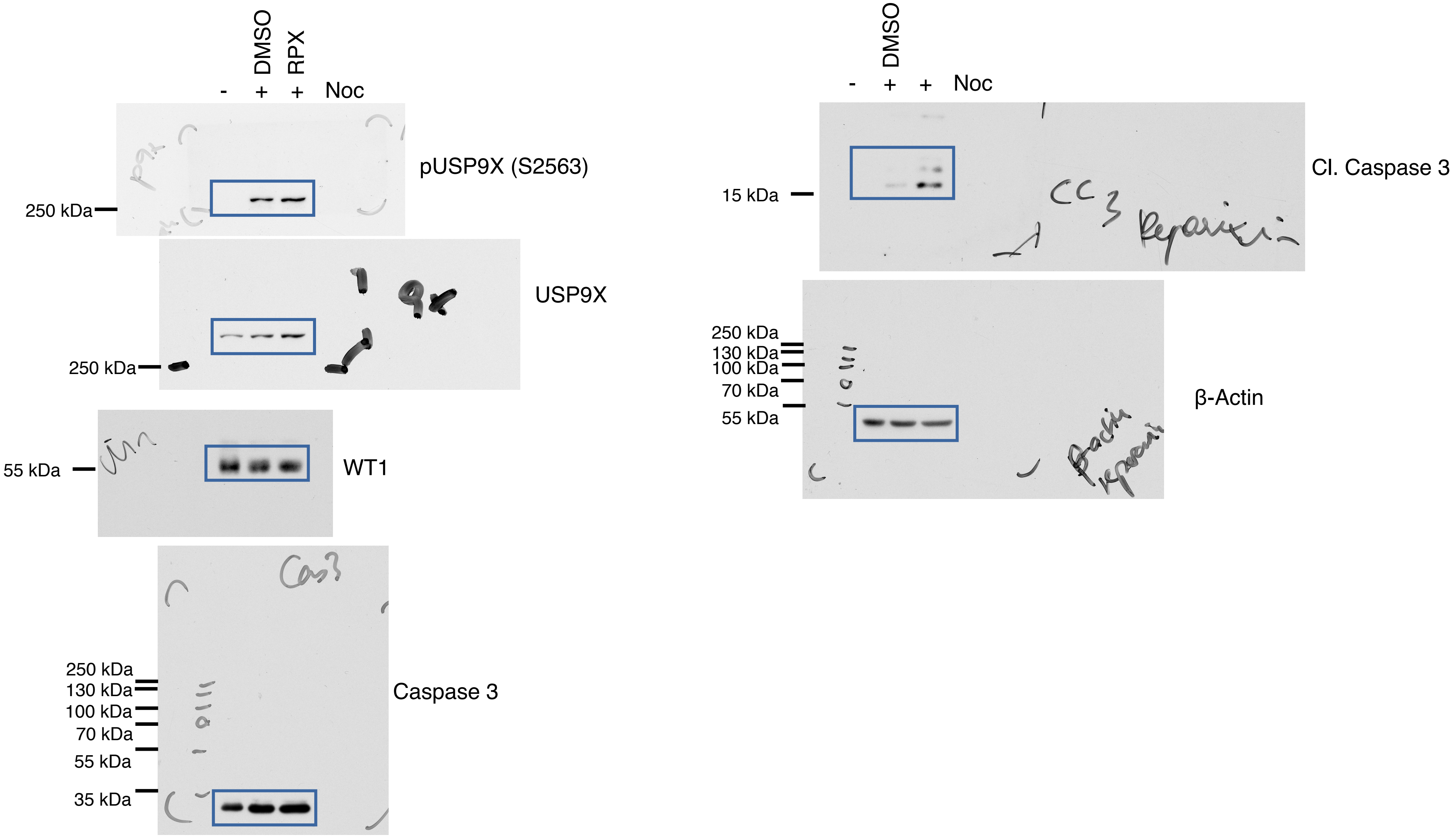
DMSO
RPX
- + + Noc
pUSP9X (S2563)
250 kDa
USP9X
250 kDa
55 kDa
WT1
250 kDa
130 kDa
100 kDa
70 kDa
55 kDa
35 kDa
Caspase 3
DMSO
- + + Noc
15 kDa
Cl. Caspase 3
250 kDa
130 kDa
100 kDa
70 kDa
55 kDa
β-Actin

Figure 4c

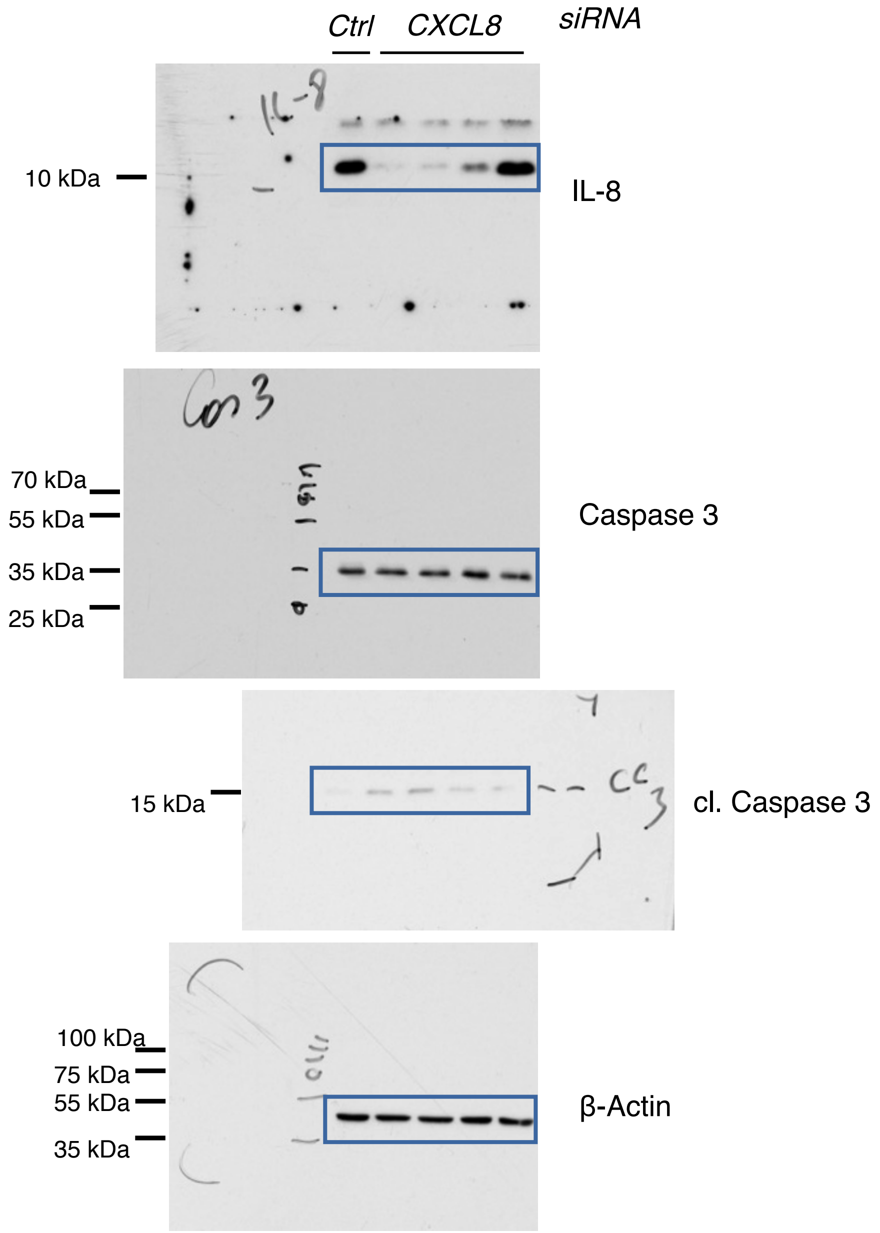
Ctrl
CXCL8
siRNA
10 kDa
IL-8
70 kDa
55 kDa
35 kDa
25 kDa
Caspase 3
15 kDa
cl. Caspase 3
100 kDa
75 kDa
55 kDa
35 kDa
β-Actin

Figure 4e

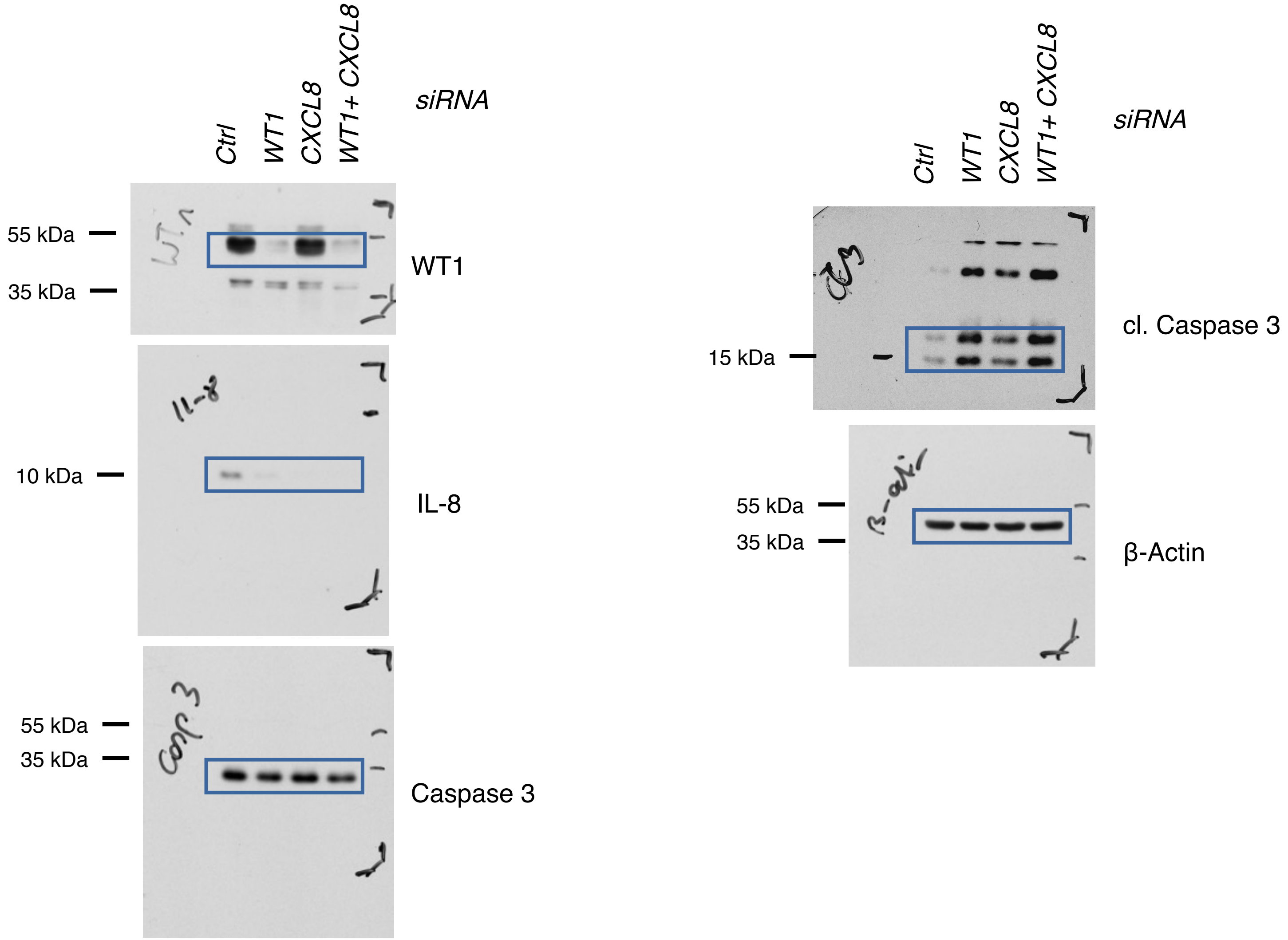

Ctrl
WT1
CXCL8
WT1+ CXCL8
siRNA
55 kDa
35 kDa
WT1
10 kDa
IL-8
55 kDa
35 kDa
Caspase 3
Ctrl
WT1
CXCL8
WT1+ CXCL8
siRNA
15 kDa
cl. Caspase 3
55 kDa
35 kDa
β-Actin

Figure 4f

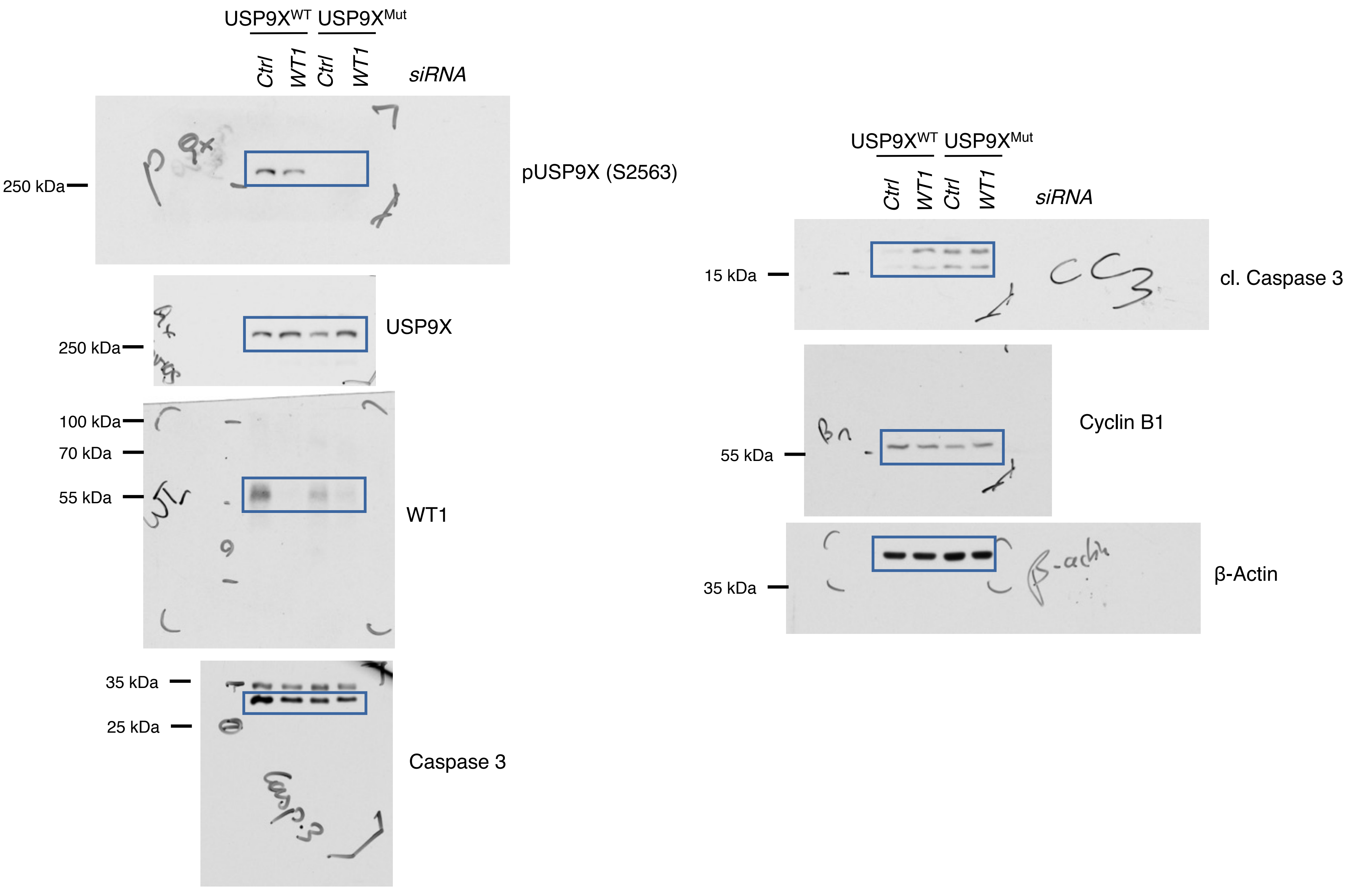

USP9XWT USP9XMut
Ctrl WT1 Ctrl WT1 siRNA
250 kDa
pUSP9X (S2563)
250 kDa
USP9X
100 kDa
70 kDa
55 kDa
WT1
35 kDa
25 kDa
Caspase 3
USP9XWT USP9XMut
Ctrl WT1 Ctrl WT1 siRNA
15 kDa
cl. Caspase 3
Cyclin B1
55 kDa
β-Actin
35 kDa

Figure 4g

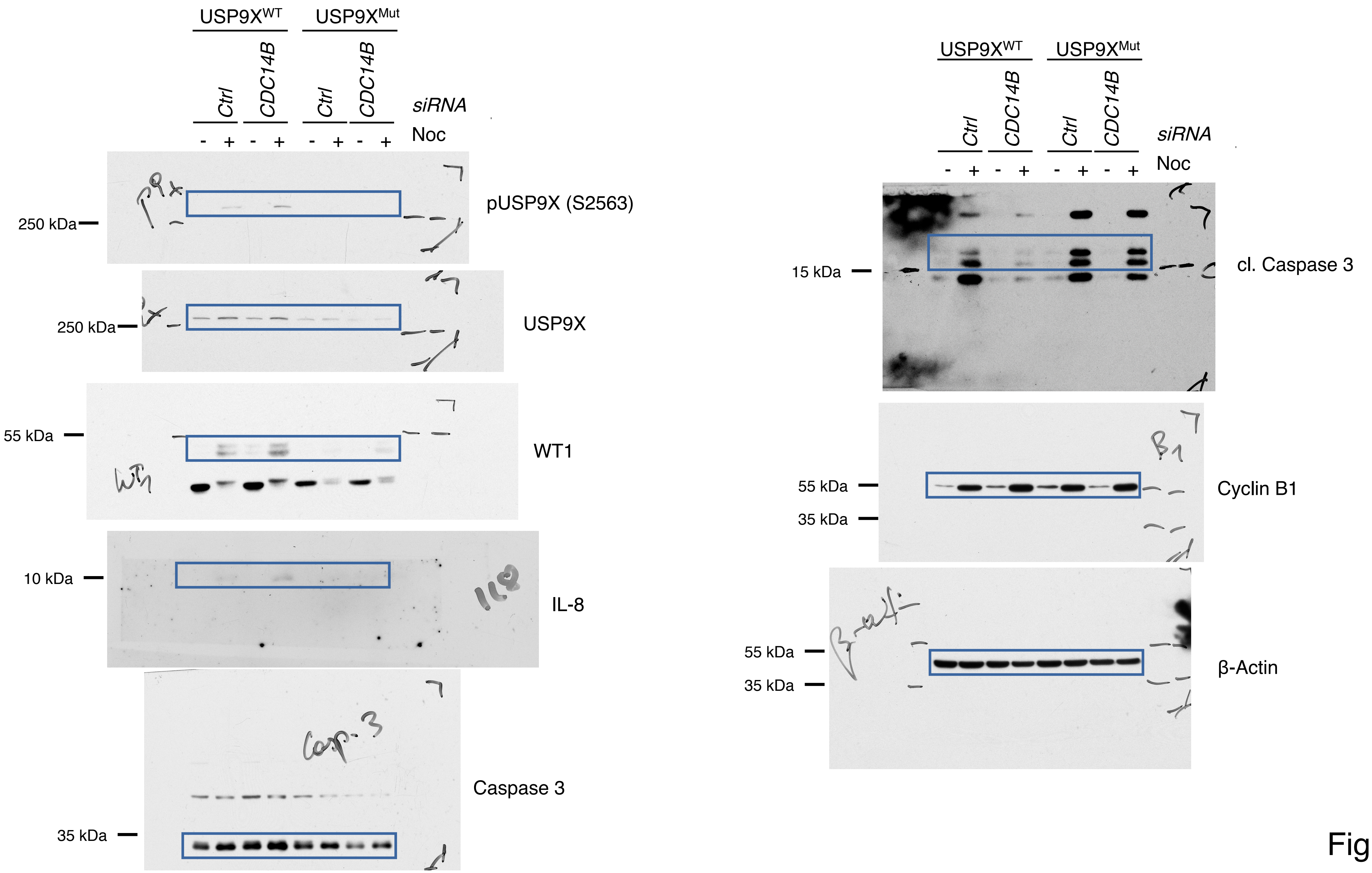
USP9X^WT
USP9X^Mut
Ctrl
CDC14B
siRNA
Noc
- + - + - + - +
250 kDa
pUSP9X (S2563)
250 kDa
USP9X
55 kDa
WT1
10 kDa
IL-8
35 kDa
Caspase 3
15 kDa
cl. Caspase 3
55 kDa
35 kDa
Cyclin B1
55 kDa
35 kDa
β-Actin

Figure 4i

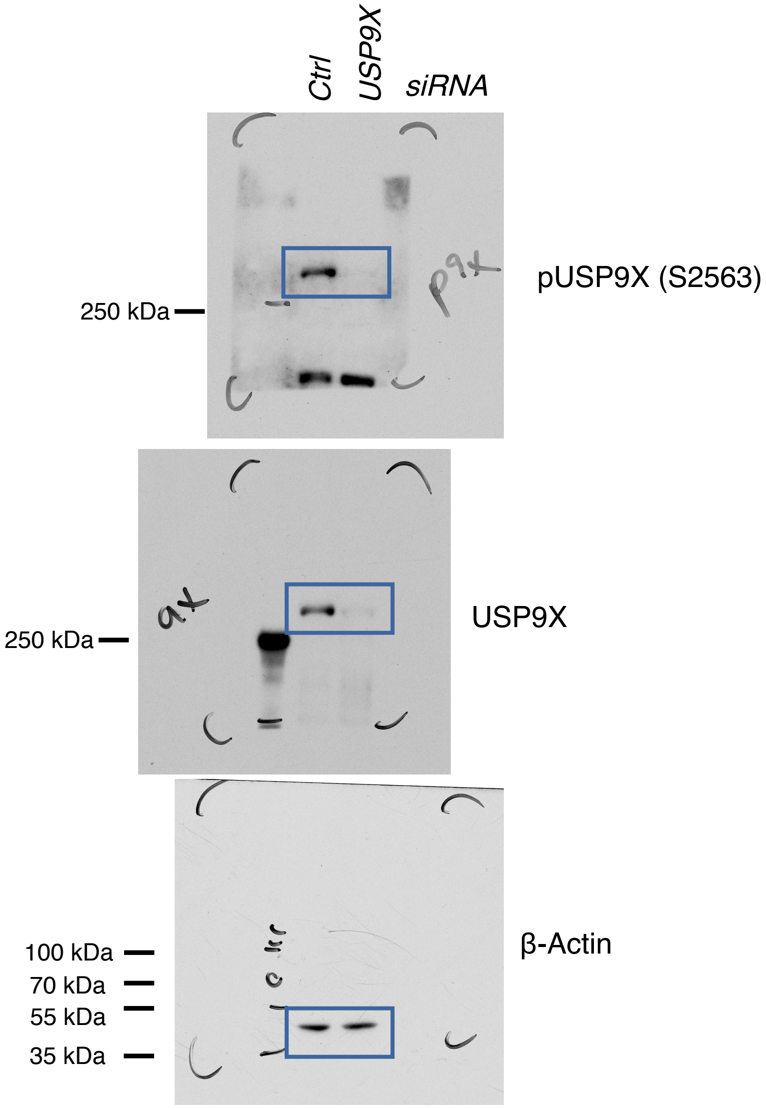

Supplementary Fig. 1d

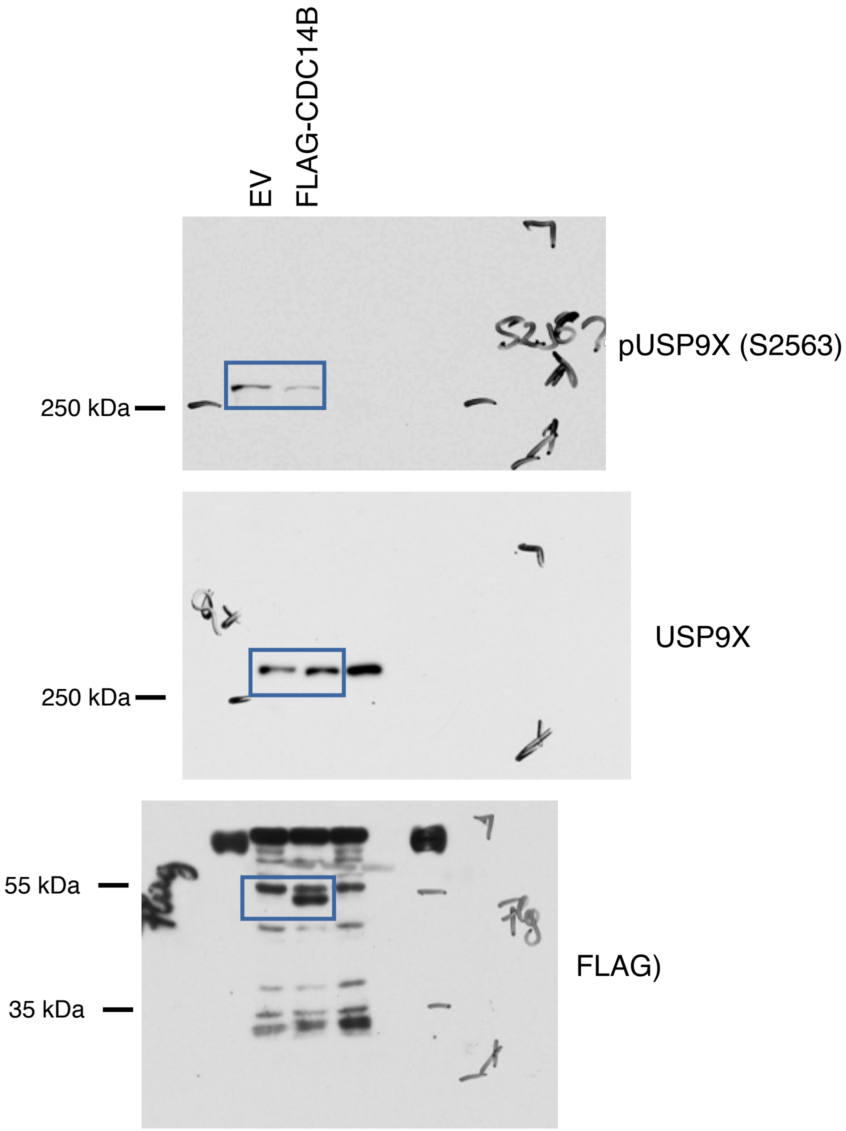

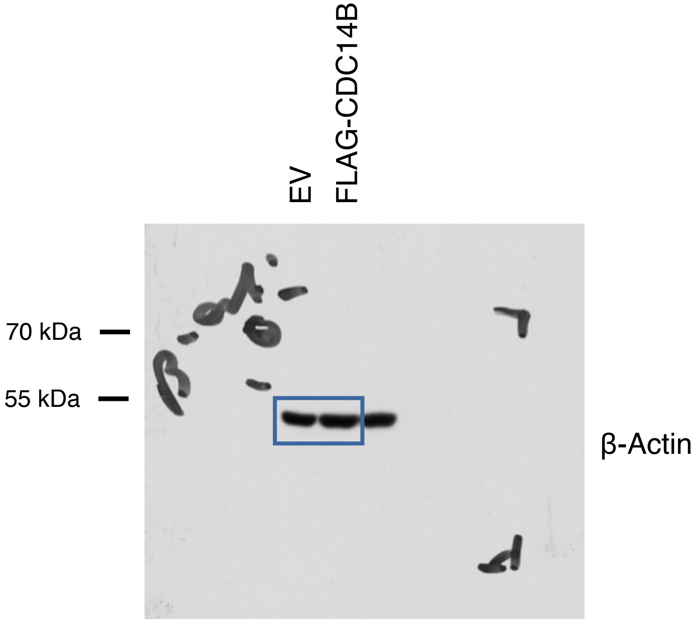

Supplementary Fig. 1f

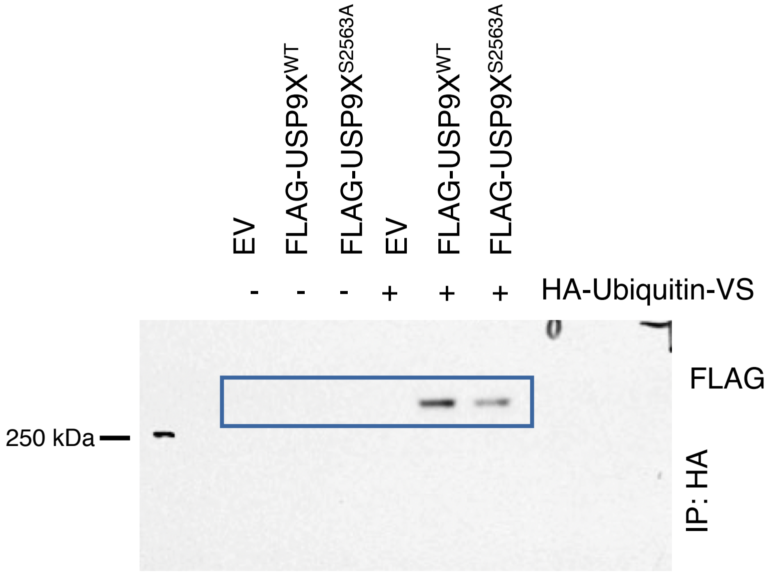

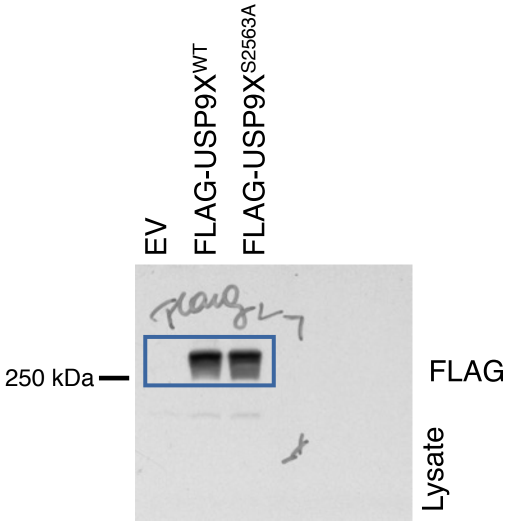

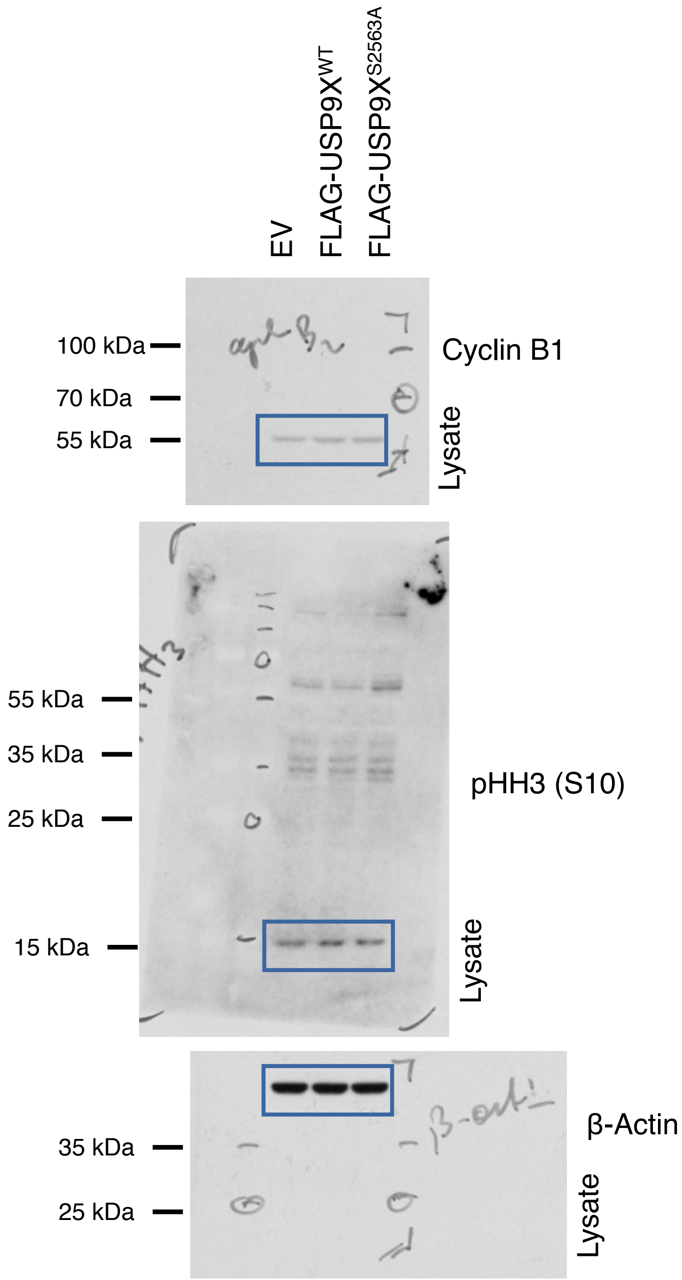

Supplementary Fig. 1h

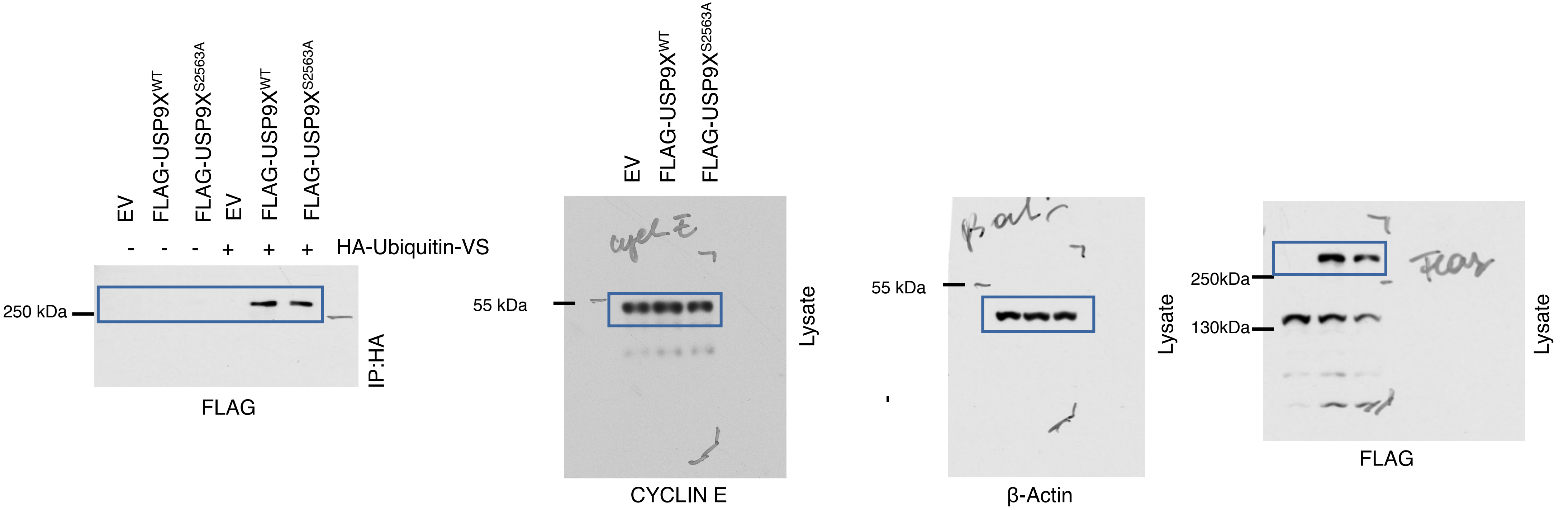

Supplementary Fig. 1i

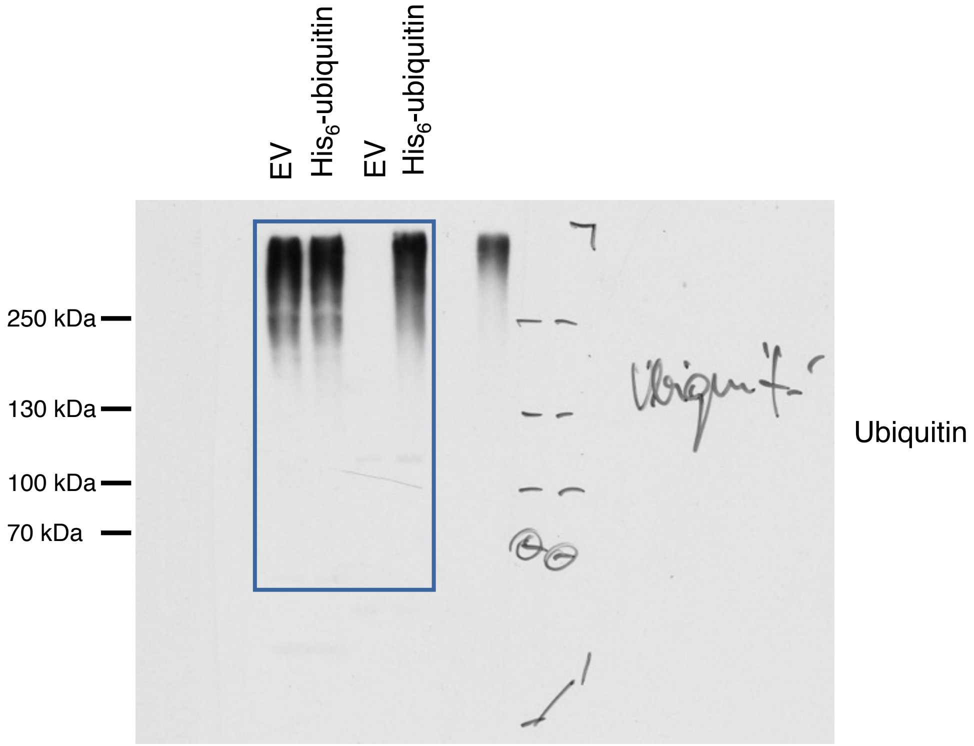

Supplementary Fig. 2b

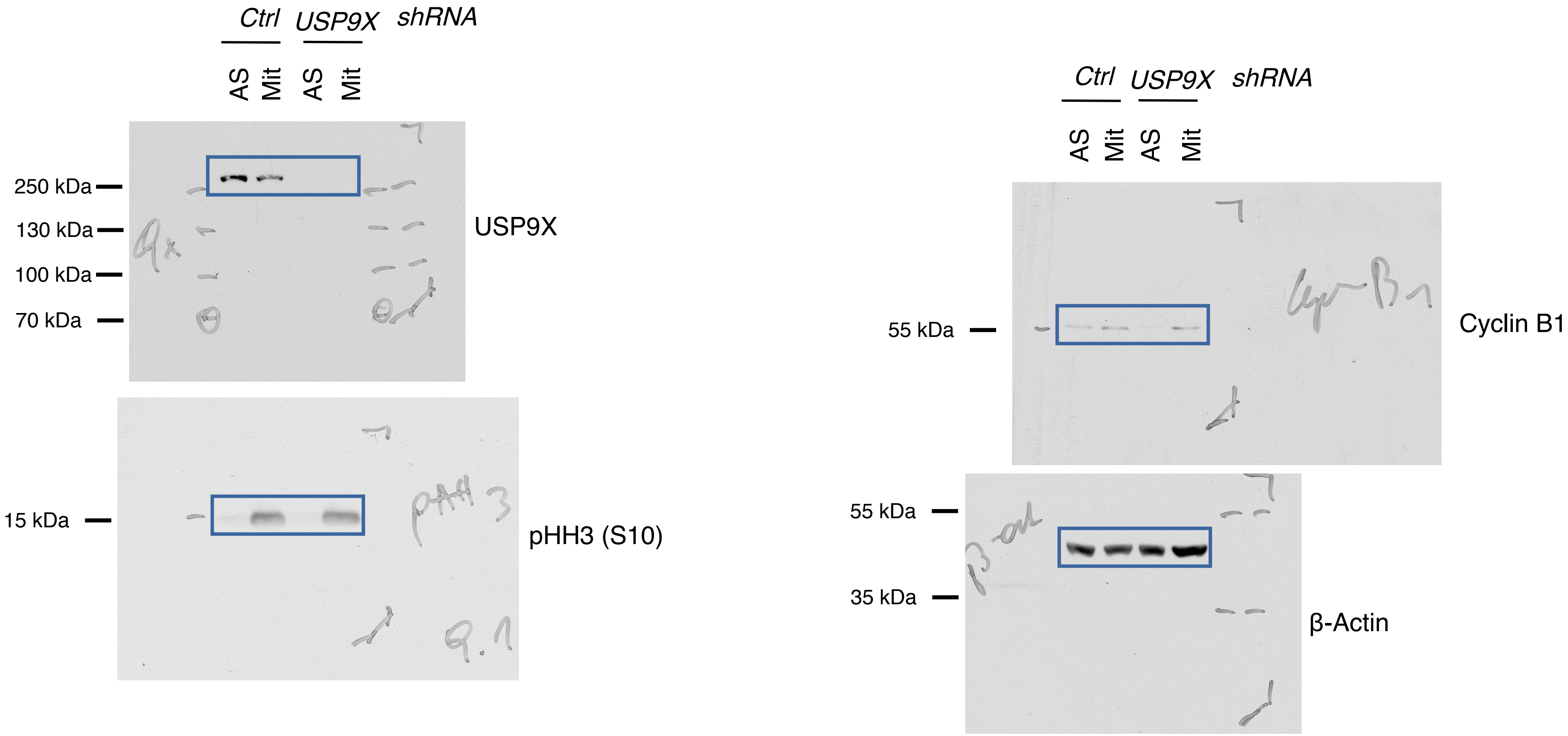

Supplementary Fig. 2c

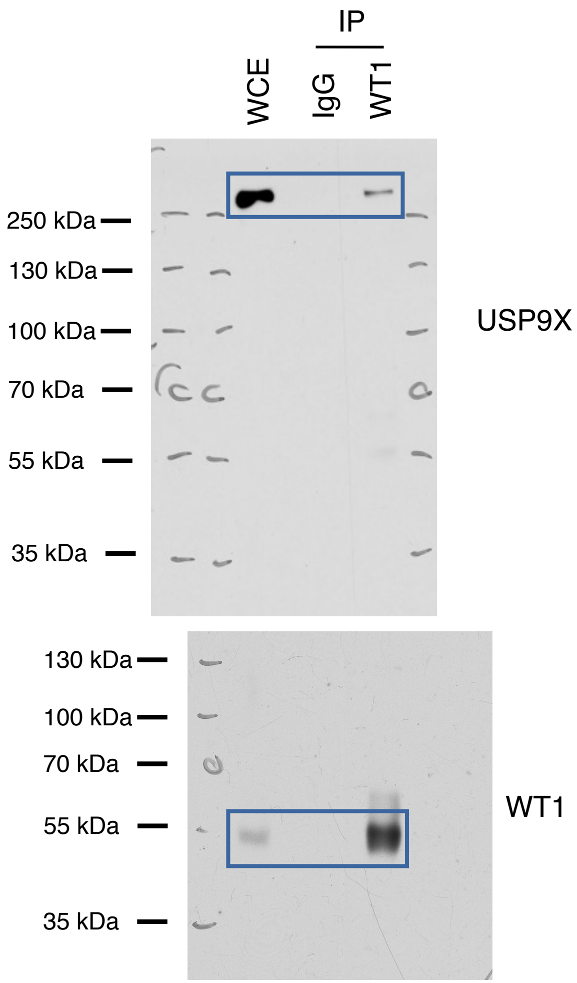

Supplementary Fig. 3a

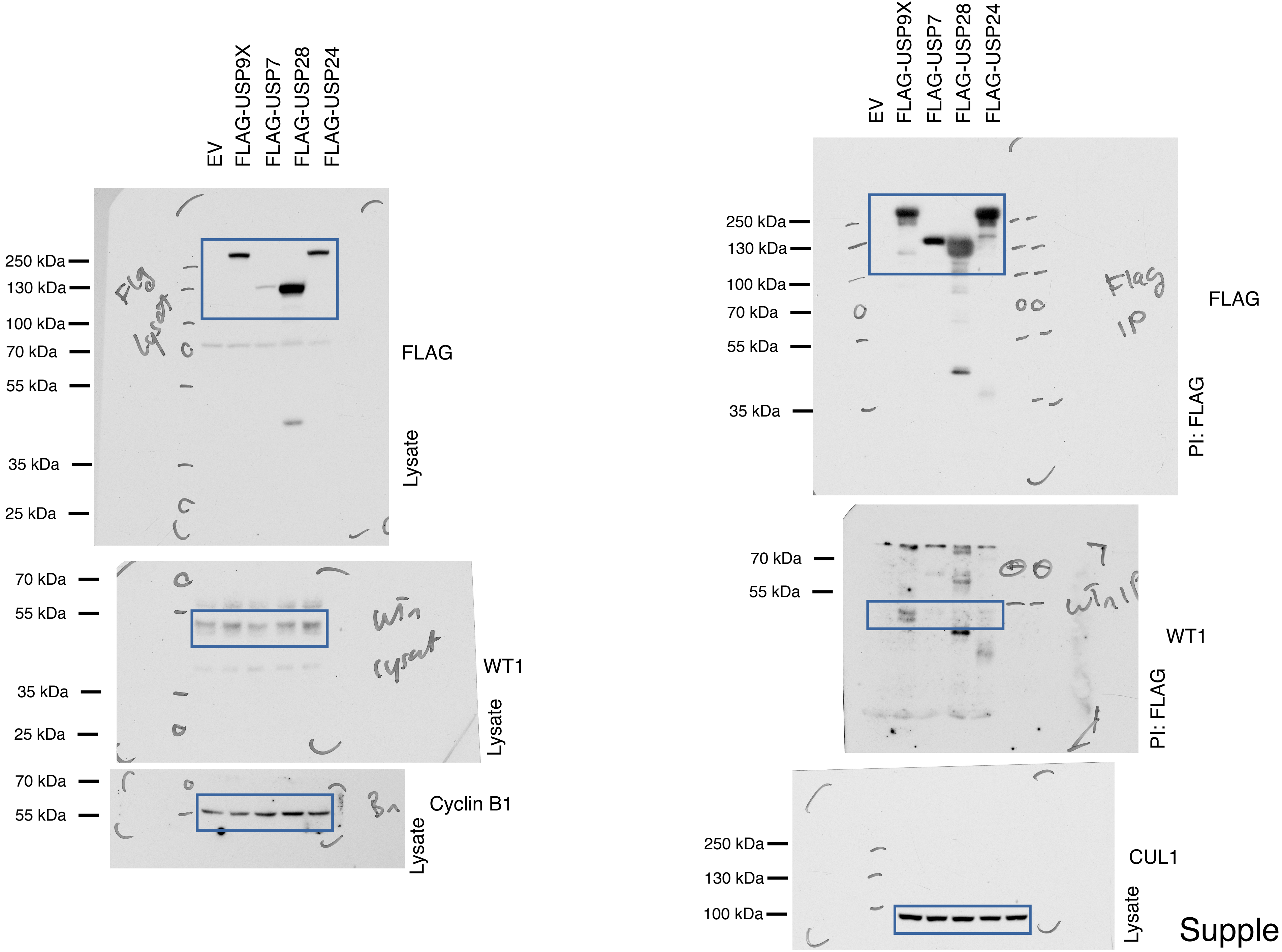

Supplementary Fig. 3b

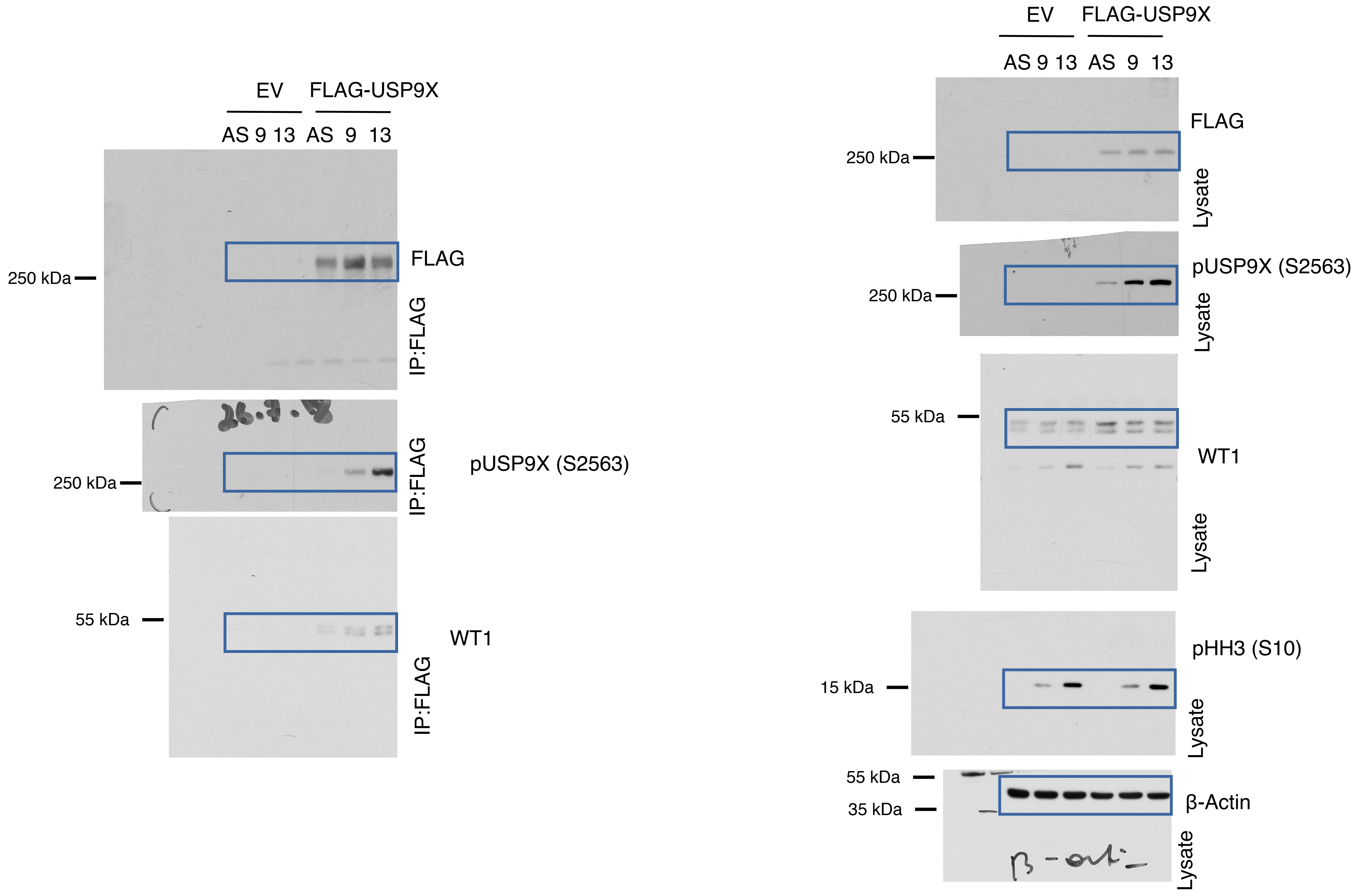

Supplementary Fig. 3c

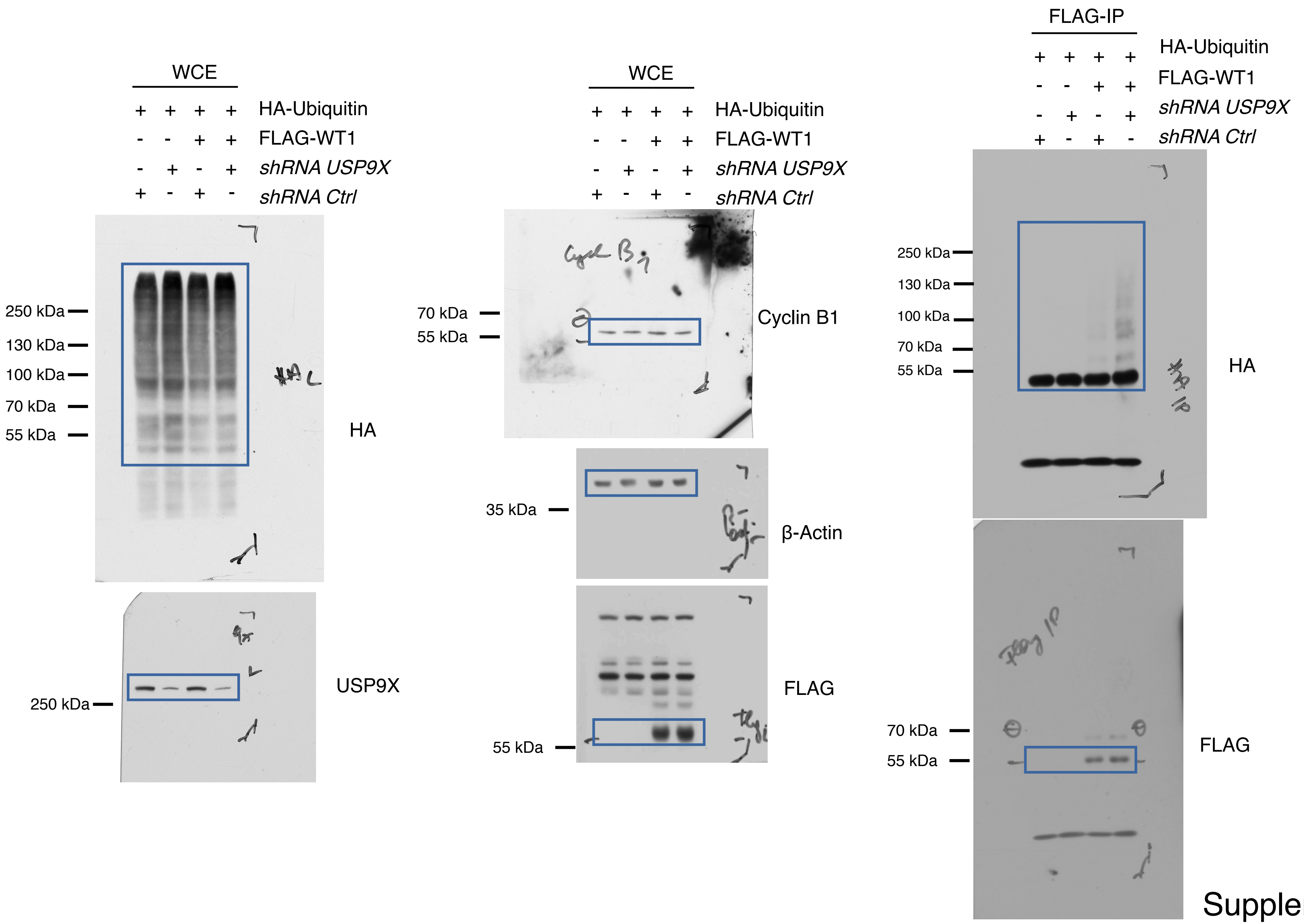

Supplementary Fig. 3e

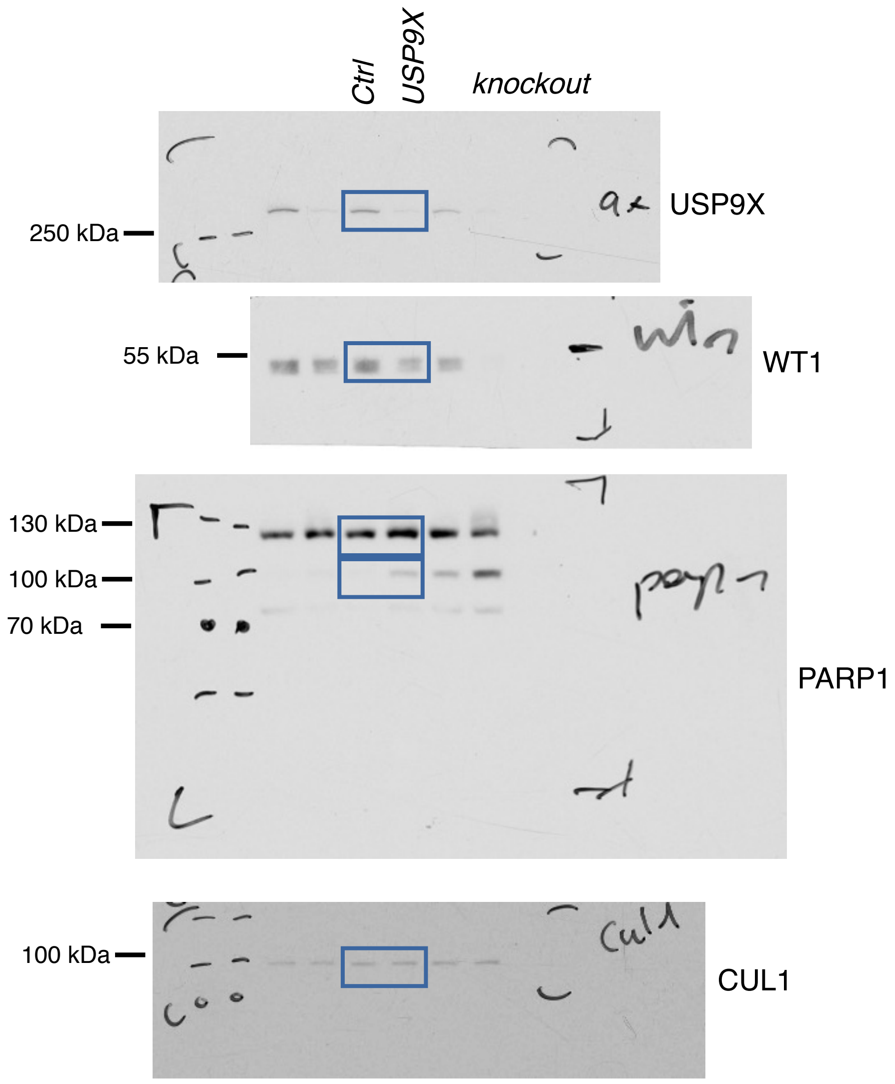

Supplementary Fig. 3f

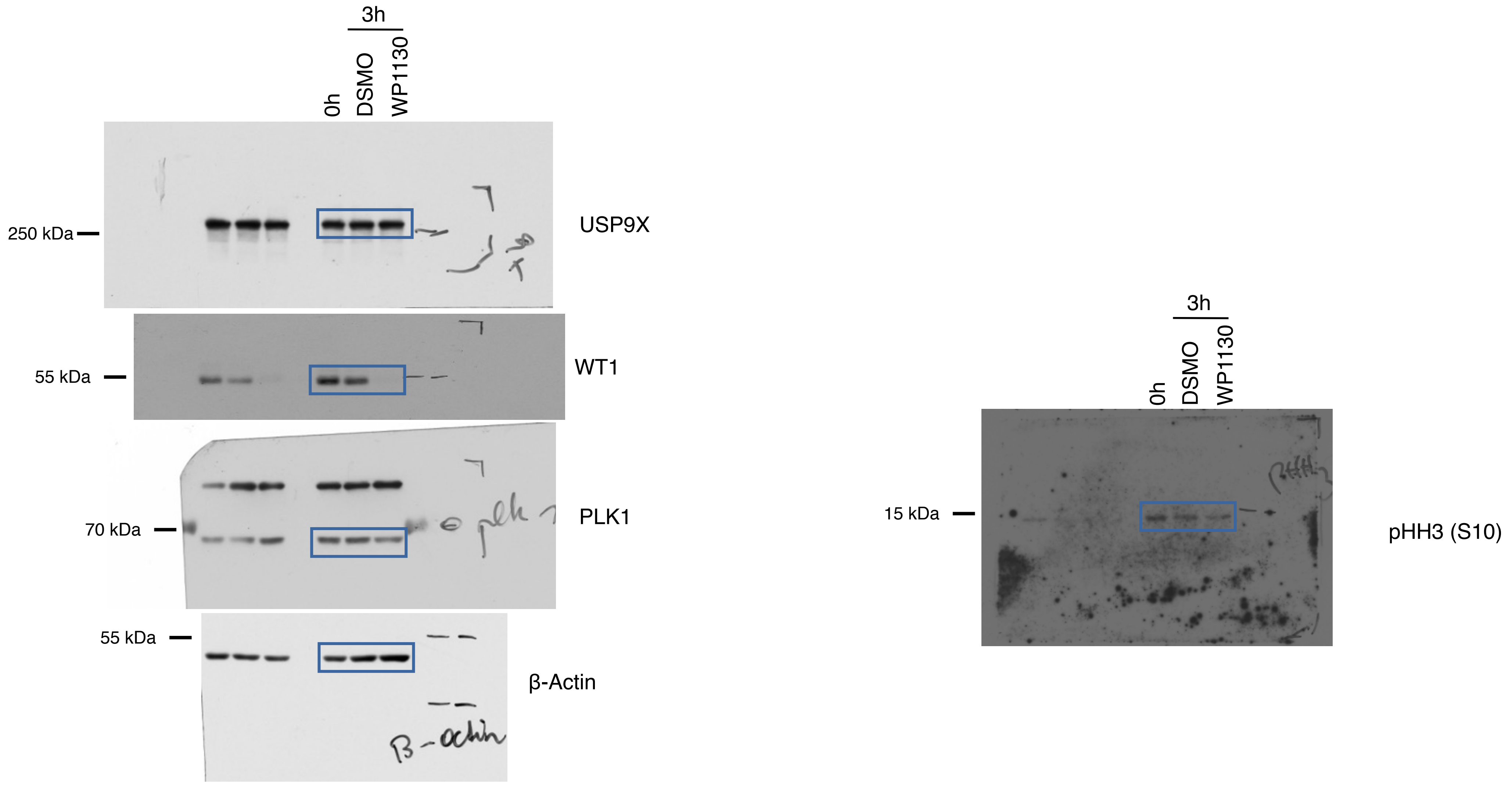

Supplementary Fig. 3h

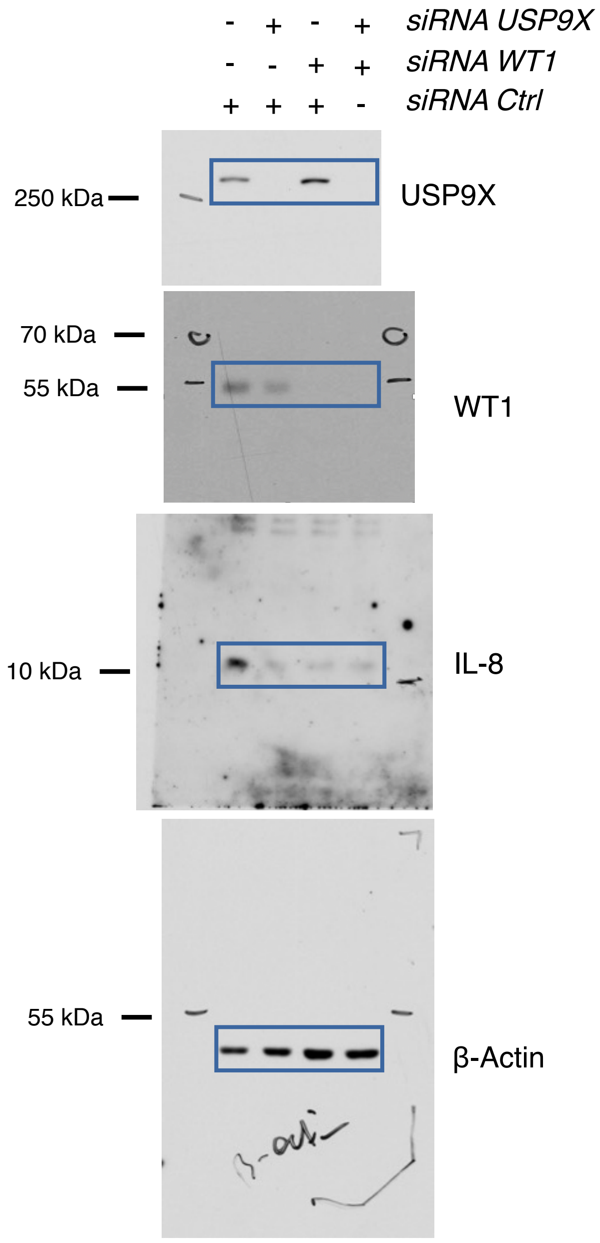

Supplementary Fig. 4d

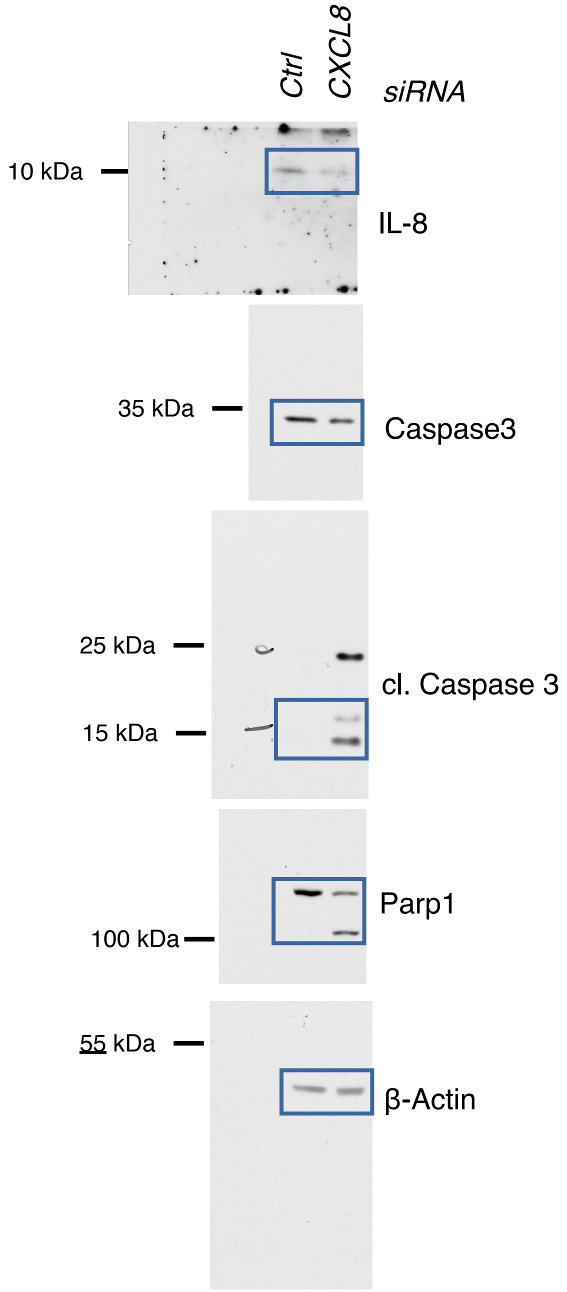

Supplementary Fig. 5a

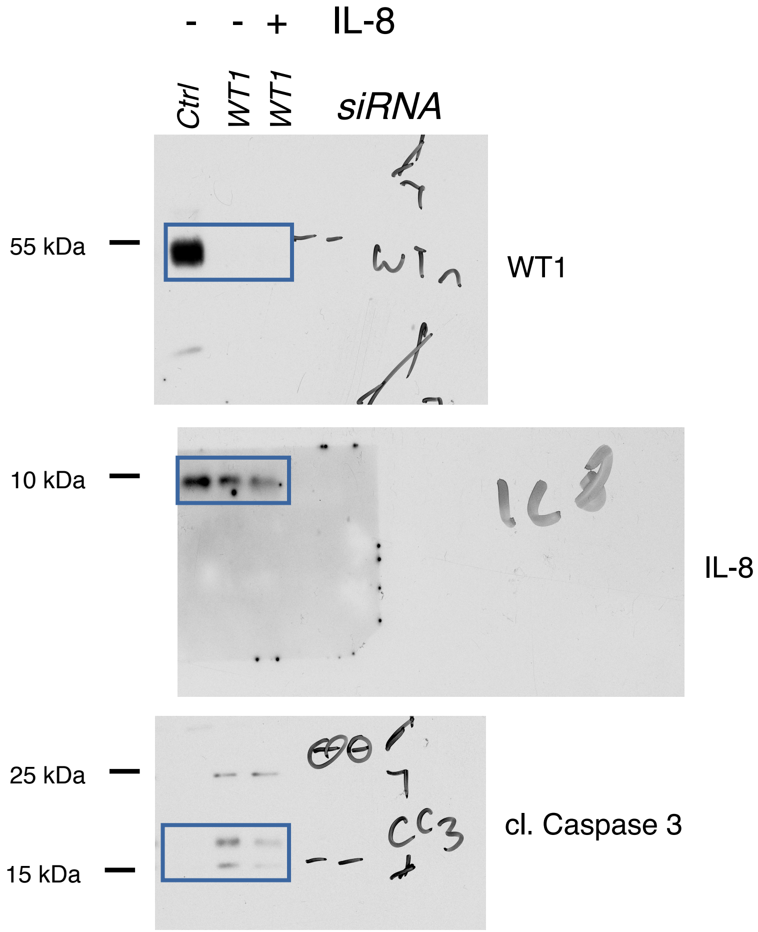

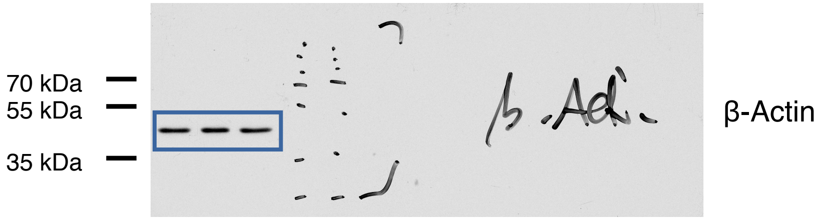

caspase3

Supplementary Fig. 5b

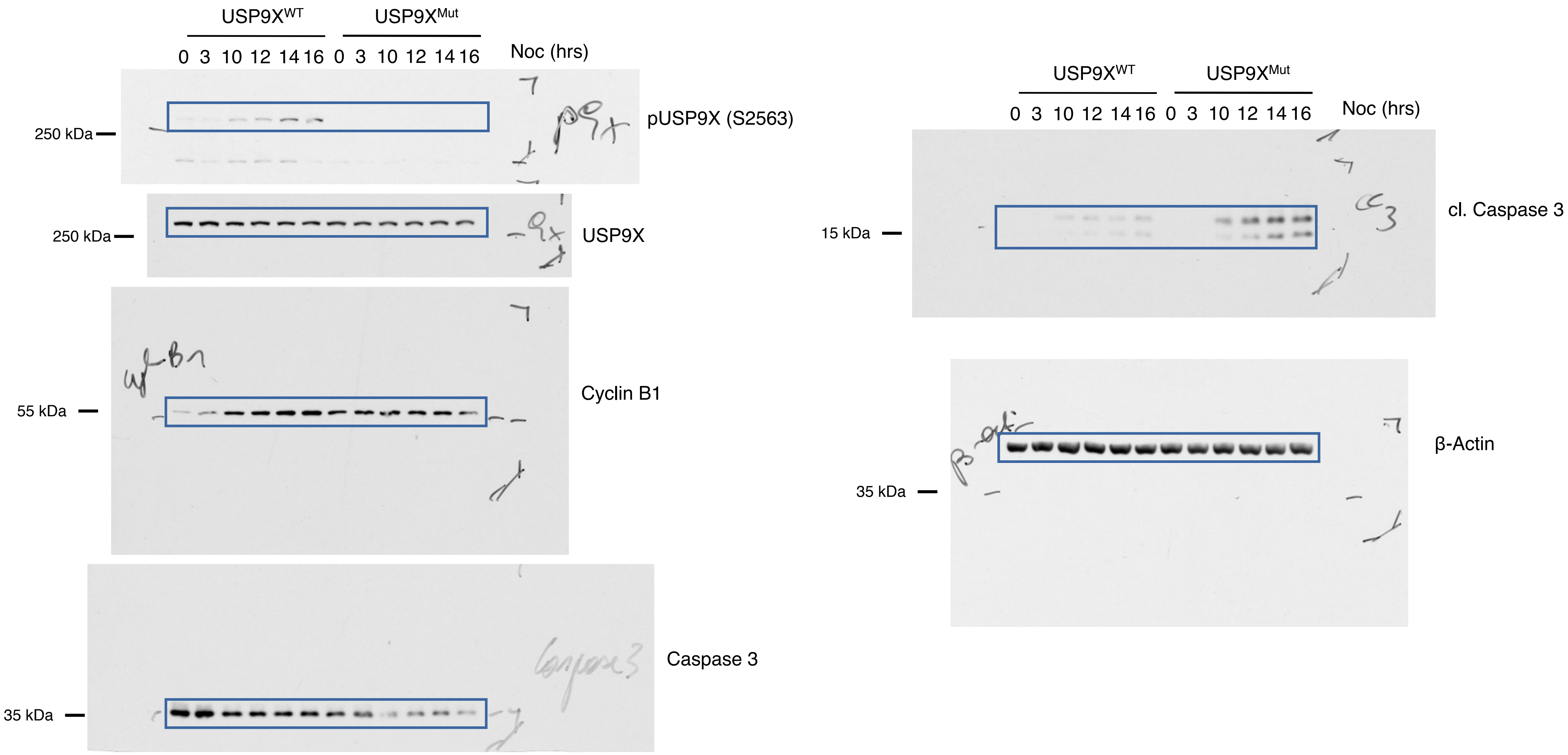

Supplementary Fig. 5f

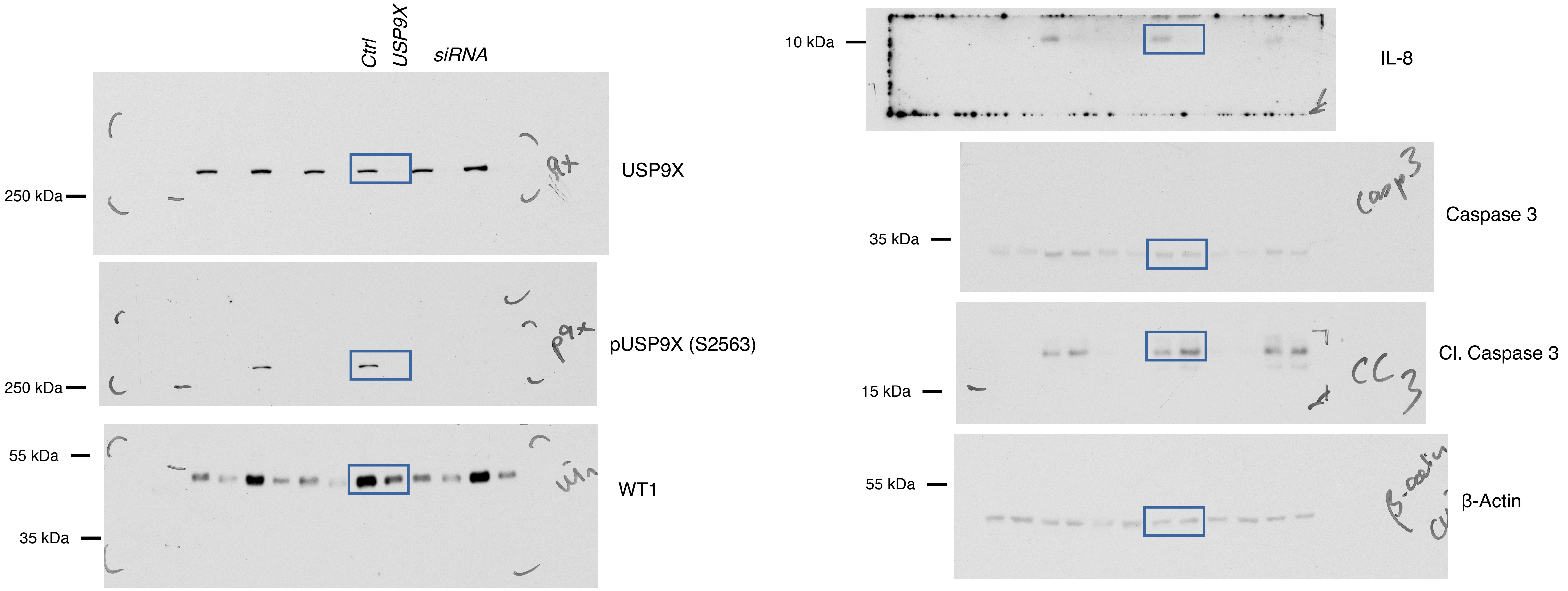

Supplementary Fig. 5g

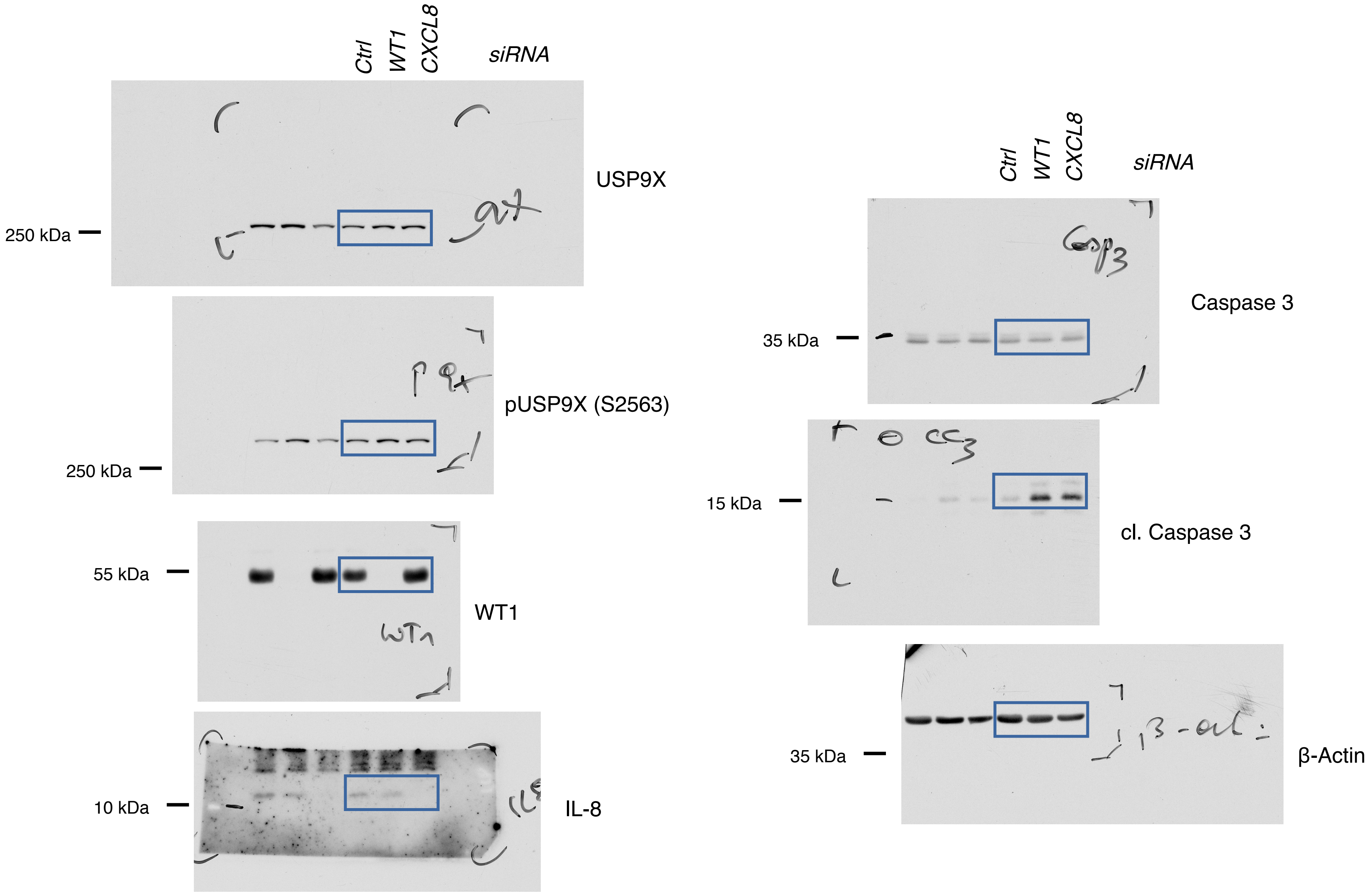

Supplementary Fig. 5h
